# The Influence of *FUT2* and *FUT3* Polymorphisms and Nasopharyngeal Microbiome on Respiratory Infections in Breastfed Bangladeshi Infants from the Microbiota and Health Study

**DOI:** 10.1128/mSphere.00686-21

**Published:** 2021-11-10

**Authors:** Aristea Binia, Léa Siegwald, Shamima Sultana, Maya Shevlyakova, Gregory Lefebvre, Francis Foata, Séverine Combremont, Aline Charpagne, Karine Vidal, Norbert Sprenger, Mahbubar Rahman, Albert Palleja, Aron C. Eklund, Henrik Bjørn Nielsen, Harald Brüssow, Shafiqul Alam Sarker, Olga Sakwinska

**Affiliations:** a Nestlé Research, Lausanne, Switzerland; b International Centre for Diarrheal Diseases Research, Bangladesh (icddr,b), Dhaka, Bangladesh; c Clinical Microbiomics, Copenhagen, Denmark; Baylor College of Medicine

**Keywords:** FUT2, microbiome, respiratory infections, secretor status

## Abstract

Acute respiratory infections (ARIs) are one of the most common causes of morbidity and mortality in young children. The aim of our study was to examine whether variation in maternal *FUT2* (α1,2-fucosyltransferase 2) and *FUT3* (α1,3/4-fucosyltransferase 3) genes, which shape fucosylated human milk oligosaccharides (HMOs) in breast milk, are associated with the occurrence of ARIs in breastfed infants as well as the influence of the nasopharyngeal microbiome on ARI risk. Occurrences of ARIs were prospectively recorded in a cohort of 240 breastfed Bangladeshi infants from birth to 2 years. Secretor and Lewis status was established by sequencing of *FUT2/3* genes. The nasopharyngeal microbiome was characterized by shotgun metagenomics, complemented by specific detection of respiratory pathogens; 88.6% of mothers and 91% of infants were identified as secretors. Maternal secretor status was associated with reduced ARI incidence among these infants in the period from birth to 6 months (incidence rate ratio [IRR], 0.66; 95% confidence interval [CI], 0.47 to 0.94; *P = *0.020), but not at later time periods. The nasopharyngeal microbiome, despite precise characterization to the species level, was not predictive of subsequent ARIs. The observed risk reduction of ARIs among infants of secretor mothers during the predominant breastfeeding period is consistent with the hypothesis that fucosylated oligosaccharides in human milk contribute to protection against respiratory infections. However, we found no evidence that modulation of the nasopharyngeal microbiome influenced ARI risk.

**IMPORTANCE** The observed risk reduction of acute respiratory infections (ARIs) among infants of secretor mothers during the predominant breastfeeding period is consistent with the hypothesis that fucosylated oligosaccharides in human milk contribute to protection against respiratory infections. Respiratory pathogens were only weak modulators of risk, and the nasopharyngeal microbiome did not influence ARI risk, suggesting that the associated protective effects of human milk oligosaccharides (HMOs) are not conveyed via changes in the nasopharyngeal microbiome. Our observations add to the evidence for a role of fucosylated HMOs in protection against respiratory infections in exclusively or predominantly breastfed infants in low-resource settings. There is no indication that the nasopharyngeal microbiome substantially modulates the risk of subsequent mild ARIs. Larger studies are needed to provide mechanistic insights on links between secretor status, HMOs, and risk of respiratory infections.

## INTRODUCTION

Acute respiratory infections (ARIs) are a leading cause of morbidity and mortality in early childhood (1). Infant feeding plays a decisive role in modifying susceptibility to ARIs; notably, the absence of breastfeeding is associated with a higher risk of infection, in particular in low-resource settings during early infancy (2–4). Human milk oligosaccharides (HMOs) are the third most abundant solid component of breast milk (5, 6). They have been hypothesized to be one of the key milk components contributing to its protective effect regarding respiratory infections (7, 8).

HMO content of breast milk is known to vary. In particular, FUT2 (α1,2-fucosyltransferase 2) and FUT3 (α1,3/4-fucosyltransferase 3) enzymes are needed for production of α1-2 and α1-3/4 fucosylated HMOs, respectively (9). *FUT2/3*-inactivating genetic variants, present in up to 30% of the population, lead to nonsecretor/Lewis-negative phenotypes which considerably influence breast milk composition and result in differences in overall quantity and concentrations of individual HMOs (10, 11). Breast milk of nonsecretor (FUT2-negative) individuals does not contain α1-2 fucosylated HMOs, for example 2′ fucosyllactose (2′FL), which is the most abundant HMO in secretor milk, or lacto-*N*-fucopentaose I and III (LNFP-I and LNFP-III). On the other hand, HMOs such as lacto-*N*-fucopentaose II (LNFP-II) and 3-fucosyllactose (3FL) are absent or very low in breast milk from Lewis-negative (FUT3-negative) mothers. Secretor and Lewis status thus highly correlate with both individual HMOs but also HMO milk groups (11) and can therefore be used as good proxies of HMO content when milk analysis is not feasible.

The variation in composition and concentration of HMOs has previously been associated with risk of infection in breastfed infants. In a cohort of predominantly Mexican mothers and their breastfed infants, the concentrations of 1,2-fucosylated HMOs such as 2′FL and lacto-*N*-di-fucohexaose (LNDFH-I) measured in milk were associated with a lower risk of moderate-to-severe diarrhea caused by calicivirus and Campylobacter (12, 13). A study in Gambia identified that the relative amount of LNFP-I and LNFP-III, but not 2′FL, in breast milk was associated with reduced parent-reported overall morbidity at 4 months postpartum (14). A recent randomized clinical trial examined the safety of feeding formula containing two HMOs, 2′FL and lacto-*N*-neo-tetraose (LNnT), in infants from approximately 2 weeks to 6 months of life (15). As a secondary outcome of this trial, reduced risk of lower respiratory tract infections (LRTIs) and decreased need for antibiotic and antipyretic use was observed in the group receiving HMOs compared to control (standard formula). This provided support for the hypothesis that fucosylated HMOs, among which 2′FL is the most abundant, could provide protection from respiratory infections.

One of the proposed mechanisms for enhancing protection against infections stipulates that HMOs act as decoys by binding and sequestering pathogens and facilitating their elimination, thus preventing pathogen attachment and invasion (16). This hypothesis is supported by some evidence from *in vitro* studies (17–19). While the attention has been recently more focused on the impact of HMOs on the gut microbiome and gut pathogens, earlier studies had indicated a potential effect on key respiratory pathogens (20). Further, it has been suggested that the carriage of respiratory pathogens, such as Streptococcus pneumoniae, Haemophilus influenzae, and Moraxella catarrhalis, could constitute a risk factor for subsequent respiratory infections (21). Could keystone pathogens residing in the respiratory tract of infants be influenced by breast milk HMOs delivered to the infant’s gastrointestinal tract? This may seem difficult to envisage; however, *Bifidobacterium*, a key infant gut species highly adapted to utilization of milk, has been observed at low abundance but 50% prevalence in the neonatal airway microbiome (22). This suggests that in young infants, milk could enter airways during breastfeeding and impact the nasopharyngeal niche. Therefore, the natural variation in HMO content may influence the risk of respiratory infections in breastfed infants via an effect on facultative pathogens.

Moreover, respiratory pathogens should not be considered in isolation. They form a part of the respiratory microbiome comprising pathogenic, commensal, and potentially beneficial organisms mutually interacting. Accordingly, the respiratory microbiome has been proposed to influence the susceptibility to respiratory infections, potentially through its antagonistic interactions with facultative respiratory pathogens (23).

Our study aimed to interrogate the link between maternal secretor and Lewis status, as a proxy for HMO composition in milk, and risk of acute respiratory infections in infants. Further, we evaluated whether the protective effect is at least partially conveyed via the effect of HMOs on the nasopharyngeal microbiome. Finally, we have tested the associations between maternal secretor and Lewis status and respiratory infections beyond the period of exclusive breastfeeding, as well as the impact of infant secretor and Lewis status on the risk of infections.

To test our hypotheses, we have used data from the Microbiota and Health Study (24), a prospective cohort study conducted in Dhaka, Bangladesh, featuring high breastfeeding rates and active surveillance of infectious episodes throughout infancy.

## RESULTS

### Characteristics of the study population.

The Microbiota and Health Study was designed to explore the interplay of the respiratory and gut microbiome and multiple factors, including environmental, maternal, and genetic variables in modulating the risk of respiratory and gastrointestinal infections in infants. The present analysis focused on a specific hypothesis linking genetic risk factors and the nasopharyngeal microbiome with ARIs during the predominantly exclusive breastfeeding period of early infancy. For that purpose, we selected mother-infant pairs for which the infants were breastfed up to at least 4 months of age. A total of 240 infants were included (91.0% of the original study; Vidal et al., in preparation). Table 1 summarizes the baseline characteristics of the population; 89.2% of infants were exclusively breastfed, and 10.8% were partially breastfed at 4 months of age. Nearly all (96.7%) were still at least partially breastfed at 6 months of age.

**TABLE 1:** Baseline characteristics and breastfeeding status of the study population

Variable	Category	All, *n* (%)	Secretor mothers (mSe+), *n* (%)	Nonsecretor mothers (mSe-), *n* (%)	Lewis-positive mothers, *n* (%)	Lewis-negative mothers, *n* (%)
Age of the mother (yrs)	>22	141 (58.8)	105 (56.5)	15 (62.5)	113 (57.4)	7 (53.8)
	≤22	99 (41.3)	81 (43.5)	9 (37.5)	84 (42.6)	6 (46.2)
Mode of delivery	Vaginal	178 (74.2)	140 (75.3)	17 (70.8)	148 (75.1)	9 (69.2)
	Cesarean section	62 (25.8)	46 (24.7)	7 (29.2)	49 (24.9)	4 (30.8)
Place of delivery	Health facility	103 (42.9)	77 (41.4)	10 (41.7)	81 (41.1)	6 (46.2)
	Home	137 (57.1)	109 (58.6)	14 (58.3)	116 (58.9)	7 (53.8)
Season at time of delivery	Premonsoon (dry) hot (Mar–May)	79 (32.9)	64 (34.4)	3 (12.5)	64 (32.5)	3 (23.1)
	Rainy monsoon (Jun–Oct)	103 (42.9)	77 (41.4)	15 (62.5)	85 (43.1)	7 (53.8)
	Cool dry winter (Nov–Feb)	58 (24.2)	45 (24.2)	6 (25.0)	48 (24.4)	3 (23.1)
Infant sex	Female	124 (51.7)	92 (49.5)	15 (62.5)	103 (52.3)	4 (30.8)
	Male	116 (48.3)	94 (50.5)	9 (37.5)	94 (47.7)	9 (69.2)
No. of siblings	Not available^*a*^	3 (1.3)	3 (1.6)		3 (1.5)	
	0: no sibling	97 (40.4)	77 (41.4)	8 (33.3)	77 (39.1)	8 (61.5)
	1: ≥1 sibling(s)	140 (58.3)	106 (57.0)	16 (66.7)	117 (59.4)	5 (38.5)
Gestational age (wks)	0: ≥37 (at term)	223 (92.9)	172 (92.5)	23 (95.8)	182 (92.4)	13 (100.0)
	1: <37 (preterm)	17 (7.1)	14 (7.5)	1 (4.2)	15 (7.6)	
Infant birth wt (kg)	Not available^*a*^	4 (1.7)	4 (2.2)		4 (2.0)	
	0: >2.5	167 (69.6)	134 (72.0)	14 (58.3)	138 (70.1)	10 (76.9)
	1: ≤2.5	69 (28.8)	48 (25.8)	10 (41.7)	55 (27.9)	3 (23.1)
Infant secretor status	Not available^*a*^	32 (13.3)	3 (1.6)		3 (1.5)	
	Secretors (iSe+)	191 (79.6)	173 (93.0)	17 (70.8)	179 (90.9)	11 (84.6)
	Nonsecretors	17 (7.1)	10 (5.4)	7 (29.2)	15 (7.6)	2 (15.4)
Infant Lewis status	Not available^*a*^	31 (12.9)	2 (1.1)		2 (1.0)	
	Lewis positive	201 (83.8)	176 (94.6)	24 (100.0)	188 (95.4)	12 (92.3)
	Lewis negative	8 (3.3)	8 (4.3)		7 (3.6)	1 (7.7)
Breastfeeding at 4 mo	Exclusively breastfed	214 (89.2)	163 (87.6)	23 (95.8)	175 (88.8)	11 (84.6)
	Partially breastfed	26 (10.8)	23 (12.4)	1 (4.2)	22 (11.2)	2 (15.4)
Breastfeeding at 6 mo	Not available^*a*^	7 (2.9)	2 (1.1)		2 (1.0)	
	Exclusively breastfed	172 (71.7)	134 (72.0)	21 (87.5)	146 (74.1)	9 (69.2)
	Partially breastfed	60 (25.0)	49 (26.3)	3 (12.5)	48 (24.4)	4 (30.8)
	Not breastfed	1 (0.4)	1 (0.5)		1 (0.5)	
Breastfeeding >6 mo	No	8 (3.3)	2 (1.1)		2 (1.0)	
	Yes	232 (96.7)	184 (98.9)	24 (100.0)	195 (99.0)	13 (100.0)
**Totals**		**240 (100.0)**	**186 (100.0)**	**24 (100.0)**	**197 (100.0)**	**13 (100.0)**

a The information could not be collected or sample not analyzed.

### Incidence of acute respiratory infections (ARIs).

**FIG 1:**
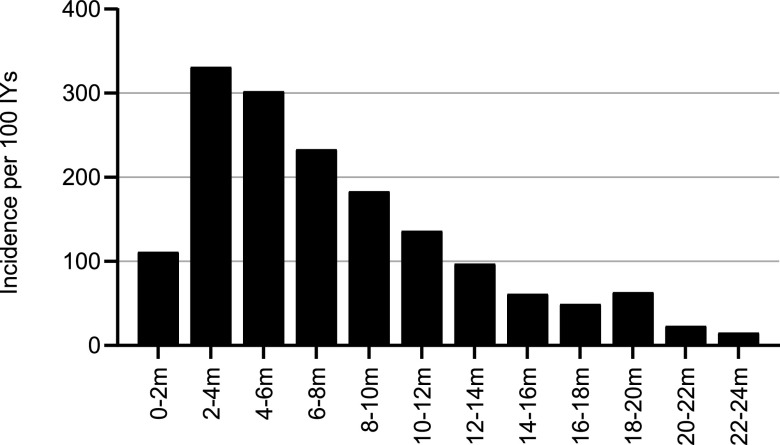
Incidence of acute respiratory infections (ARIs) by bi-monthly intervals. Incidence is expressed as the number of episodes per 100 infant-years of observation.

A total of 644 episodes of ARI were recorded during the follow-up period of 24 months. ARI was defined as one or more of the following symptoms: cough, runny nose, nasal congestion, ear discharge, and rapid breathing. The number of infants decreased from an initial *n* = 264 to *n* = 204 at 24 months, and data for 240 infants were available from birth to 6 months. The peak incidence of ARI was observed between 2 and 6 months of age, with 263 episodes (Fig. 1).

### Characterization of *FUT2* and *FUT3* secretor- and Lewis-defining status variants.

*FUT2* and *FUT3* exome sequences were obtained for 213 mothers and 212 infants in order to identify missense/nonfunctional single-nucleotide polymorphisms (SNPs) known to affect the secretor and Lewis status, as described in Materials and Methods. After quality control, 19 variants for the mothers and 18 variants for the infants passed the filters, including 7 missense SNPs in the total population which defined secretor and Lewis status (Table S1).

10.1128/mSphere.00686-21.6 TABLE S1 Summary of the genotyping results in the population. Download Table S1, DOCX file, 0.03 MB. Copyright © 2021 Binia et al. 2021 Binia et al. https://creativecommons.org/licenses/by/4.0/ This content is distributed under the terms of the Creative Commons Attribution 4.0 International license.

In order to define secretor status, we considered all missense *FUT2* SNPs known to result in a nonfunctional enzyme (11, 25–27). In our study population, three *FUT2* missense SNPs (rs601338, rs1047781, and rs200157007) were present. Missense SNP rs602662 was in perfect linkage disequilibrium with rs601338; thus, we considered it redundant in our selection (data not shown). Based on these three SNPs, 24 mothers (11.4%) and 17 infants (8.2%) were identified as nonsecretors (Se–). Similarly, according to four missense *FUT3* SNPs present in the study population (rs3745635, rs28362459, rs3894326, and rs812936) (11) and used to define the Lewis (positive/negative) status, 13 mothers (6.2%) and 8 infants (3.8%) were identified as Lewis negative (Le–) (Table 1). Only two mothers were both *FUT2* and *FUT3* negative. No significant differences were observed for any of the maternal and infant variables between secretors and nonsecretors, as well Lewis positive compared to Lewis negative (Fisher’s exact text, χ^2^ test).

### Maternal secretor status was associated with lower ARI risk in infants.

**FIG 2:**
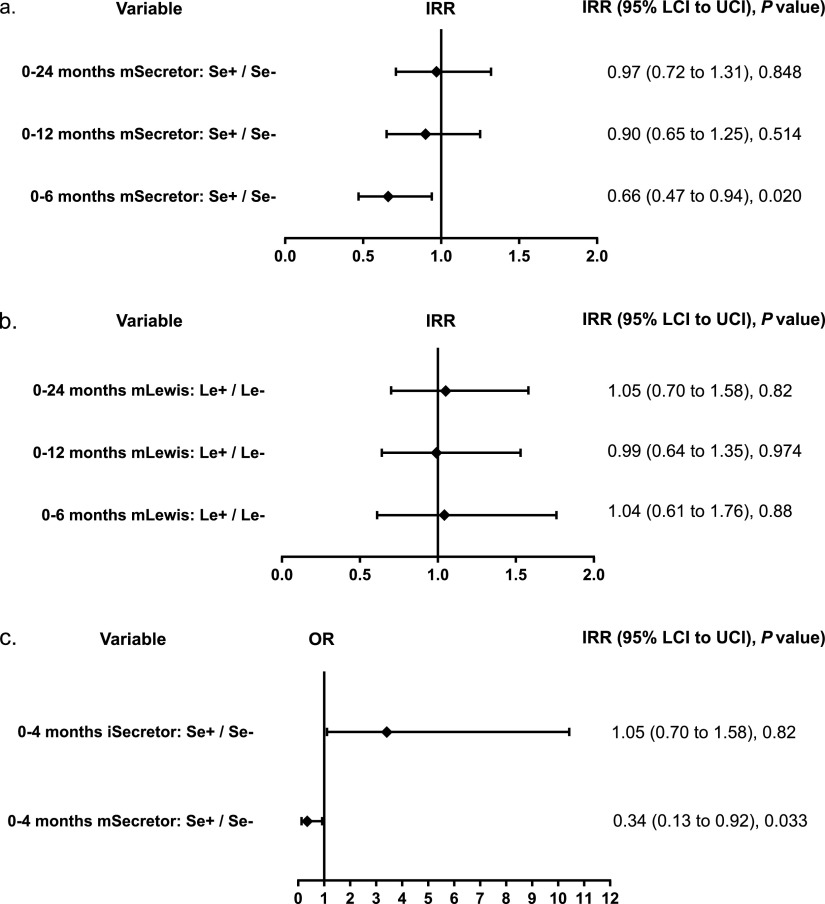
Forest plots for IRR or OR (95% lower confidence interval [LCI] to upper confidence interval [UCI]) represent ARI risk in relation to maternal secretor status (mSecretor) from 0 to 24 months (a), Lewis maternal status (mLewis) from 0 to 24 months (b), and maternal and infant secretor status (mSecretor and iSecretor) from 0 to 4 months (c). Status could be either positive (Se+, Le+) or negative (Se–, Le–). The negative status was considered a reference category. These results derive from the regression model considering both maternal and infant secretor/Lewis status.

Infants of secretor-positive mothers (mSe+) had a significantly lower incidence of ARI than those of secretor-negative mothers (mSe–) during the first 6 months of life, with an incidence rate ratio (IRR) of 0.66 (95% confidence interval [CI]. 0.47 to 0.94; *P = *0.020) (Fig. 2a). Maternal Lewis status was not associated with ARI incidence during the first 6 months of life (Fig. 2b). No significant associations were found between maternal secretor or Lewis status and incidence of ARI in infants during later time points (Fig. 2a and b).

We conducted additional *post hoc* analyses focusing on the nearly exclusive breastfeeding period coinciding with the peak of ARIs (Fig. 1). The statistical model employing IRR did not converge for the period of 0 to 4 months. Therefore, the analyses over this period were performed using the occurrence of at least one ARI episode, employing odds ratios (OR). As observed for the IRR of ARIs during the first 6 months, infants of secretor-positive mothers (mSe+) had a lower ARI occurrence in the period of 0 to 4 months (Fig. 2c), with an OR of 0.34 (95% CI, 0.13 to 0.92; *P = *0.033).

Regarding the infection susceptibility linked to an infants’ secretor and Lewis status, exploratory analysis employing OR for the 0- to 4-month period revealed a significantly increased risk of ARIs for secretor-positive infants only, with and without accounting for the maternal secretor status (OR = 3.41; 95% CI, 1.11 to 10.43; *P = *0.031) (Fig. 2c). However, this association was not detected using IRR for the period of 0 to 6 months (IRR = 1.21; 95% CI, 0.76 to 1.92; *P = *0.45) (Fig. S1). Single SNP associations were finally tested for the number of ARI episodes, but none of the results reached the level of statistical significance after adjustment for multiple testing with a 0.05 false-discovery rate (FDR) (data not shown).

10.1128/mSphere.00686-21.3 FIG S1 Forest plots for IRR (95% CI) calculated for risk of ARI incidence in relation to infant secretor status (iSe) and Lewis status (iLe). Status could be either positive (Se+, Le+) or negative (Se–, Le–). The negative status was considered a reference category. These results derive from the regression model considering both maternal and infant secretor/Lewis status. Download FIG S1, DOCX file, 0.03 MB. Copyright © 2021 Binia et al. 2021 Binia et al. https://creativecommons.org/licenses/by/4.0/ This content is distributed under the terms of the Creative Commons Attribution 4.0 International license.

### Nasopharyngeal colonization by respiratory pathogens.

**FIG 3:**
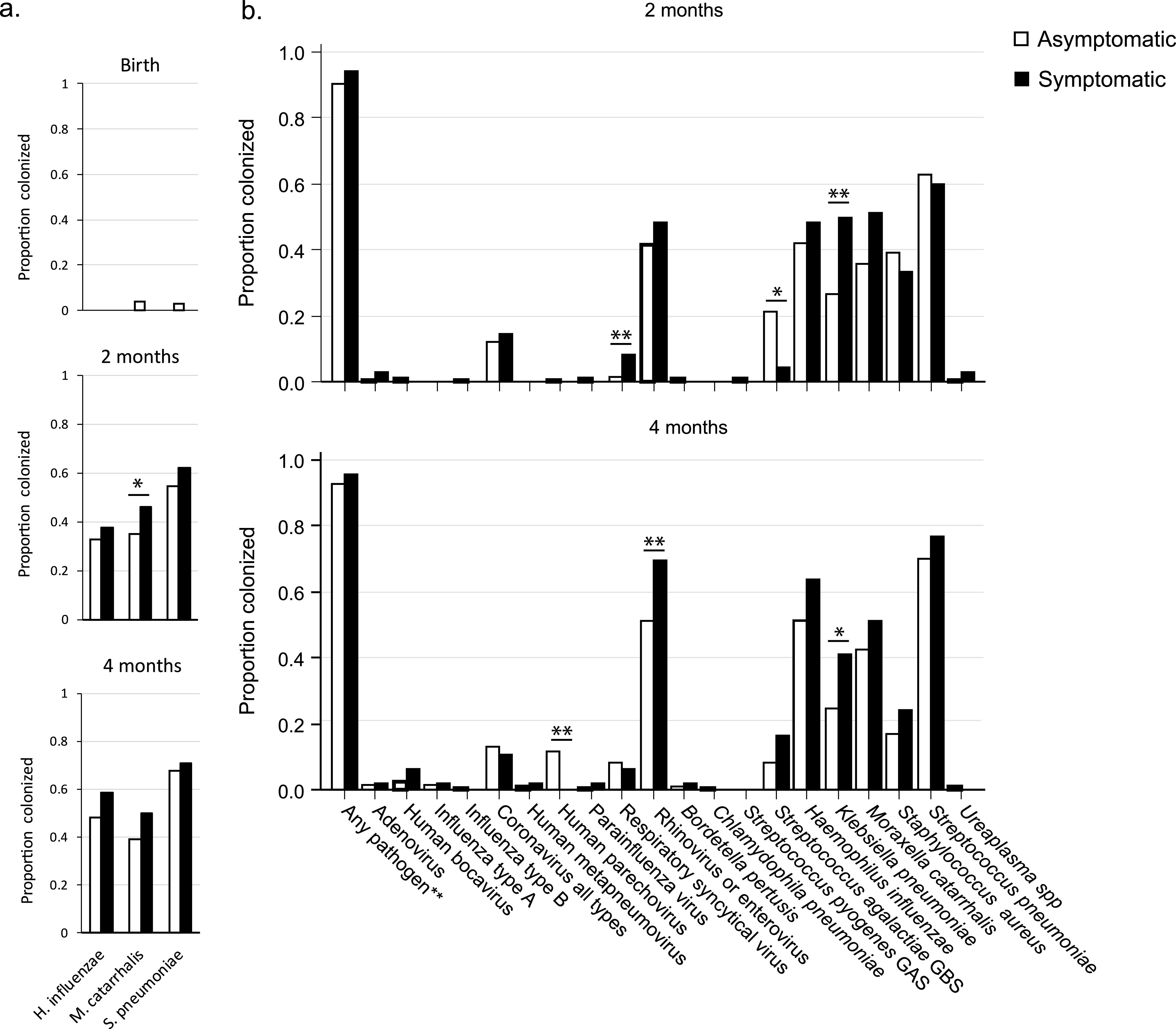
Nasopharyngeal colonization pathogens in infants asymptomatic and symptomatic for ARIs at the time of sample collection assessed by culture (a) and TaqMan array cards (b). One star (*) denotes a trend (0.05 < *P* < 0.1) and ** denotes a significant (*P < *0.05) difference between symptomatic and asymptomatic infants.

To examine the hypothesis that the maternal secretor and Lewis status affected the risk of ARI in infants by modulating respiratory pathogen colonization, we examined their presence using both culture and molecular methods. A large proportion of infants were colonized by facultative bacterial respiratory pathogens, as assessed by culture (S. pneumoniae, H. influenzae, and M. catarrhalis) (Fig. 3A). Molecular detection of bacterial and viral respiratory targets at 2 and 4 months of age confirmed the frequent colonization by bacterial pathogens (Fig. 3B). In addition to S. pneumoniae, H. influenzae, and M. catarrhalis, colonization with Klebsiella pneumoniae and Staphylococcus aureus was common. Among viruses, rhinovirus was the most frequently detected, notably in asymptomatic infants showing 42% prevalence at 2 months of age that increased to 53% at 4 months of age.

As expected, symptomatic infants tended to have a higher risk of being colonized, when assessed by both culture and molecular methods (Fig. 3). However, the effect size was small, with a trend for more prevalent colonization significant only for K. pneumoniae among bacterial pathogens. Regarding viruses, rhinovirus was significantly more common in symptomatic infants at 4 months of age, with the same trend at 2 months. Respiratory syncytial virus was also overrepresented in symptomatic infants at 2 months, although this trend was no longer significant at 4 months. Surprisingly, human parechovirus was more often detected in asymptomatic infants at 4 months, but not at 2 months (Fig. 3B).

### Effect of secretor and Lewis status on nasopharyngeal colonization by facultative bacterial respiratory pathogens.

Infants of Lewis-positive mothers were less likely to be colonized by M. catarrhalis in the period from birth to 6 months (OR = 0.42; CI, 0.22 to 0.83; *P = *0.036), and there was a trend for less colonization by H. influenzae for infants of secretor-positive mothers in the same period (OR = 0.55; CI; 0.29 to 1.05; *P = *0.083; only detectable when infant secretor status was included in the model). No other significant associations were observed.

### Nasopharyngeal colonization by facultative bacterial respiratory pathogens in asymptomatic infants was weakly associated with subsequent ARIs.

**FIG 4:**
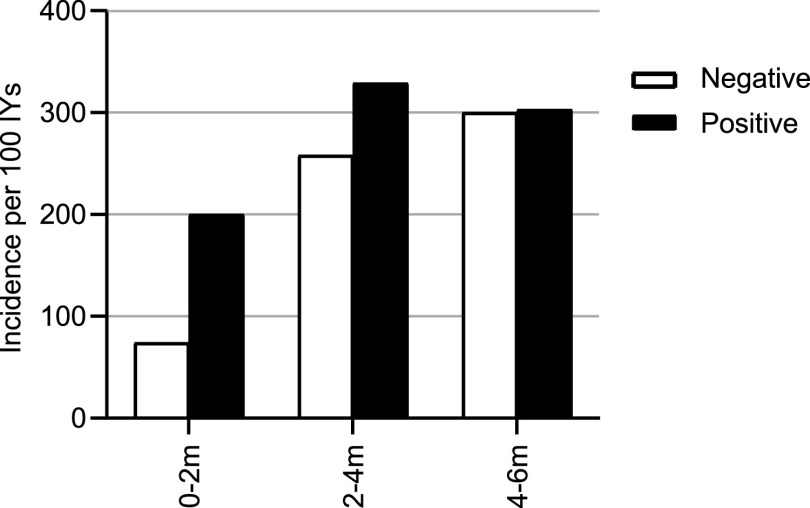
ARI incidence in the 2-month period following the sample collection for asymptomatic infants colonized (positive) by facultative bacterial respiratory pathogens (M. catarrhalis, S. pneumoniae, H. influenzae) and those who were not colonized by these pathogens (negative) at the beginning of the observation period.

The data on colonization by facultative pathogens were collected at bi-monthly intervals; therefore, the influence of the colonization status on risk of subsequent ARIs was analyzed using a repeated measures model. The selection of covariates for this analysis was based on the identification of the significant predictors of ARIs in the entire cohort (Vidal et al. in preparation). The colonization by facultative bacterial pathogens (M. catarrhalis, S. pneumoniae, H. influenzae) was associated with marginally higher infection risk in the following 2-month period (IRR = 1.59; CI, 1.01 to 2.53; *P = *0.047) (Fig. 4, Table S2), apparently driven by M. catarrhalis (IRR = 1.64; CI, 1.12 to 2.40; *P = *0.011) (Table S2). Although the above-described *P* values refer to the entire analysis period, numerically, the largest difference in risk was observed for the colonization at birth for the period 0 to 2 months (Fig. 4) despite low rates of colonization at birth (Fig. 3A).

10.1128/mSphere.00686-21.7 TABLE S2 Association between colonization status and risk of subsequent ARIs. Data on infants who were asymptomatic at the moment of sample collection are included. The model is adjusted for interaction of age and breastfeeding status. Download Table S2, DOCX file, 0.03 MB. Copyright © 2021 Binia et al. 2021 Binia et al. https://creativecommons.org/licenses/by/4.0/ This content is distributed under the terms of the Creative Commons Attribution 4.0 International license.

We also evaluated the impact of colonization of a wide range of bacterial and viral pathogens detected by molecular methods on cumulative ARI infection rate through a Kaplan-Meier analysis, available at 2 and 4 months. No differences were observed in cumulative ARI infection rate according to infant colonization status (Fig. S2).

10.1128/mSphere.00686-21.4 FIG S2 Kaplan-Meier analysis of pathogens positive in at least 20 subjects from (a) 2 months onward and (b) 4 months onward. Each subplot shows the association between the cumulative infection rate over time, split by each pathogen status (positive versus negative). In each subplot, the horizontal axis is the number of days since (a) 2 months and (b) 4 months, and the vertical axis is the cumulative infection rate (probability that a subject would be infected at least once by this day). Included covariates were sex, mode of delivery, season at (a) 2 months and (b) 4 months, mother secretor status, breastfed status at 6 months, weight z score at birth, and whether ARI happened before (a) 2 months and (b) 4 months. Other relevant covariates (e.g., infant secretor status or breastfeeding status at 4 months) were excluded due to extreme imbalance between groups. Download FIG S2, DOCX file, 0.5 MB. Copyright © 2021 Binia et al. 2021 Binia et al. https://creativecommons.org/licenses/by/4.0/ This content is distributed under the terms of the Creative Commons Attribution 4.0 International license.

### Characterization of the nasopharyngeal microbiome using shotgun metagenomics.

We hypothesized that the overall nasopharyngeal microbiome could significantly contribute to subsequent respiratory outcomes and potentially convey the effect of maternal secretor status. Therefore, we characterized the nasopharyngeal microbiome of infants at 2 and 4 months of age using shotgun metagenomics, focusing on asymptomatic infants to consider the microbiome as a potential cause and not as a consequence of disease.

A total of 422 samples were sequenced on the Illumina HiSeq platform with an average of 38.1 million (M) read pairs per sample. As expected for a low-biomass microbiome (28), most samples were dominated by human host reads, which were filtered out as described in Supplemental Text S1. By assembling the remaining reads and clustering the genes called from the contigs, a nonredundant nasopharyngeal microbial catalog of 3,110,772 genes was built. By using the coabundance principle across samples, 55 metagenomic species (MGS) were identified, of which 53 were annotated up to the species level. On average, 94.7% of the genes within each MGS mapped to the same reference genome, suggesting a reliable high-quality MGS taxonomy.

10.1128/mSphere.00686-21.1 TEXT S1 Nasopharyngeal microbiome sequencing and data analysis. Download Text S1, DOCX file, 0.04 MB. Copyright © 2021 Binia et al. 2021 Binia et al. https://creativecommons.org/licenses/by/4.0/ This content is distributed under the terms of the Creative Commons Attribution 4.0 International license.

**FIG 5:**
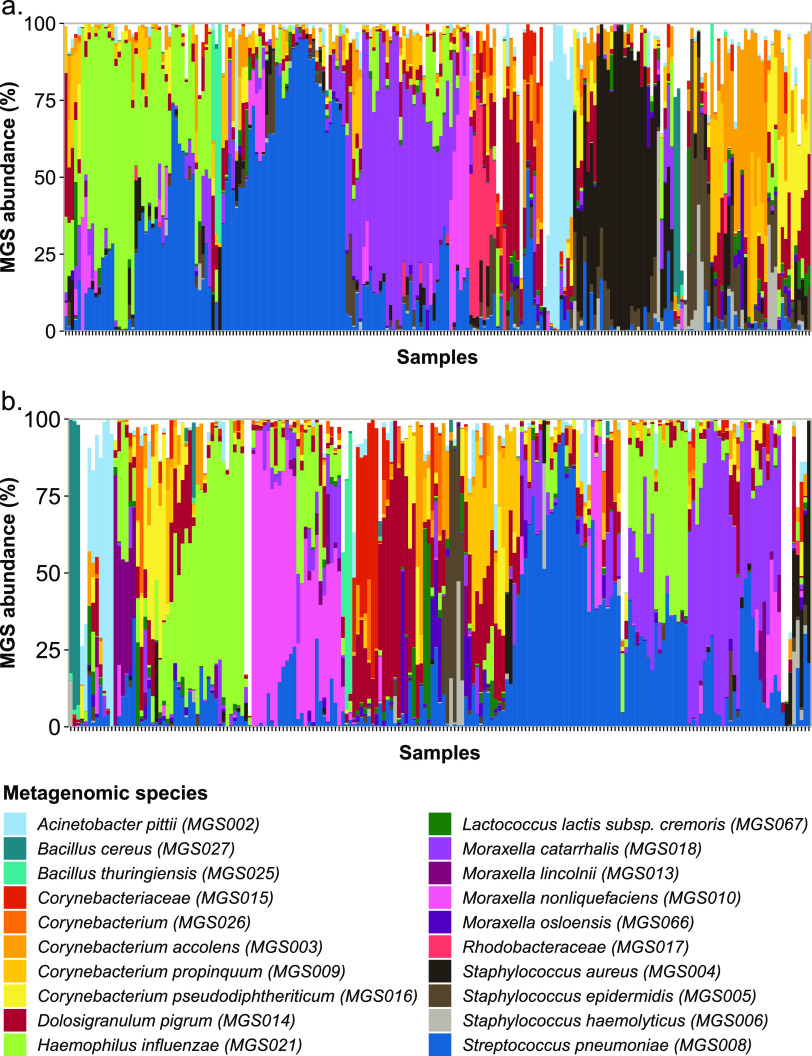
The relative abundance of the top 20 most abundant metagenomic species (MGS) in nasopharyngeal samples from (a) 2- and (b) 4-month-old asymptomatic infants. Samples were ordered according to hierarchical clustering based on Bray-Curtis dissimilarities among the samples (clustering and associated ordering is time point-specific and therefore different between panels a and b).

The observed nasopharyngeal microbiome at both time points was composed of both commensal and facultative pathogens (Fig. 5). The most abundant species included Streptococcus pneumoniae, Haemophilus influenzae, Moraxella catarrhalis, Dolosigranulum pigrum, and Staphylococcus aureus; the detailed description of MGS abundance and full taxonomical characterization can be found in the Supplemental Data Set S1.

10.1128/mSphere.00686-21.2 DATA SET S1 Relative abundance and annotation of all MGSs per sample. Abundance is the estimated proportion of nonhost cells. This is based on the number of reads aligned but also takes into account gene/genome size and read length, adjusted for multimapped reads. Download Data Set S1, XLSX file, 0.2 MB. Copyright © 2021 Binia et al. 2021 Binia et al. https://creativecommons.org/licenses/by/4.0/ This content is distributed under the terms of the Creative Commons Attribution 4.0 International license.

The differentiation among individuals was striking; the variation attributable to individuals accounted for most of the variance (permutational multivariate analysis of variance [PERMANOVA] test; *R*^2^ = 62%, *P* = 0.0005). The effect of time was much less pronounced, and no clear separation between samples of the two age groups was observed (Fig. S3), even though the shift in composition was statistically significant (PERMANOVA test; *R*^2^ = 1.5%, *P = *0.0001). MGS richness slightly decreased from 2 months (average, 8; range, 1 to 19) to 4 months (average, 7.4; range, 2 to 16) (two-sided Wilcoxon signed-rank test, *P = *0.027), while there was no difference in alpha diversity (Shannon diversity index = 1.16 and 1.11, at 2 and 4 months, respectively).

10.1128/mSphere.00686-21.5 FIG S3 Principal-coordinate analysis (PCoA) based on the Bray-Curtis dissimilarities among the microbiome MGS composition of each sample. Samples are indicated by dots color-coded by age. The mean of all the samples belonging to the same group is indicated with a larger dot (centroid), which is connected to all samples from the same group. Download FIG S3, DOCX file, 0.3 MB. Copyright © 2021 Binia et al. 2021 Binia et al. https://creativecommons.org/licenses/by/4.0/ This content is distributed under the terms of the Creative Commons Attribution 4.0 International license.

### Effect of maternal secretor and Lewis status and extrinsic factors on the nasopharyngeal microbiome.

If the protective effect of HMOs were conveyed by modulation of the infant microbiome, we would expect an association between maternal secretor and/or Lewis status and microbiome composition. This was, however, not the case: maternal secretor and Lewis status showed no significant association with the composition of the nasopharyngeal microbiome (Table 2). Other variables previously reported to influence the nasopharyngeal microbiome (such as prior infection, mode of delivery, breastfeeding, and season at sampling) showed no significant association with microbiome except season at sampling, which was more pronounced at 2 months of age.

**TABLE 2:** Influence of extrinsic factors on the nasopharyngeal microbiome at 2 and 4 months of age analyzed by PERMANOVA tests with 10,000 permutations^*a*^

Clinical factor	Df	*R* ^2^	*P*
2 mo			
Mode of delivery	1	0.010	0.065
Maternal secretor	1	0.004	0.63
Maternal Lewis	1	0.006	0.41
Infant secretor	1	0.006	0.29
Infant Lewis	1	0.005	0.57
Breastfeeding at 2 mo	2	0.007	0.90
Prior infection	1	0.005	0.55
**Season at sampling**	2	0.048	**0.0001**
4 mo			
Mode of delivery	1	0.005	0.53
Maternal secretor	1	0.003	0.90
Maternal Lewis	1	0.010	0.070
Infant secretor	1	0.009	0.12
Infant Lewis	1	0.005	0.62
Breastfeeding at 4 mo	2	0.009	0.77
Prior infection	1	0.007	0.31
**Season at sampling**	2	0.033	**0.0003**

a Significant factors are highlighted in bold. Df, degrees of freedom; *P*, *P* value in pseudo-F test.

### Nasopharyngeal microbiome composition could not explain subsequent ARI occurrence.

To evaluate whether the nasopharyngeal microbiome is linked to subsequent ARIs we employed a wide range of machine learning approaches. However, despite transforming the microbiome data in different ways, taking into account the impact of extrinsic factors on the microbiome, considering different follow-up time periods, and using different machine learning methods (Supplemental Text S1), the 72 models we built exhibited highly unsatisfactory trade-off between specificity and sensitivity, thus precluding linking the nasopharyngeal microbiome with subsequent ARIs (Table S3).

10.1128/mSphere.00686-21.8 TABLE S3 Summary of the ARI prediction models performance predicting from 2- and 4-month microbiome and phenotypic data. The “Abund data” column shows the abundance data transformation used. The “N” column shows the samples used after removing incomplete cases. The “Cov” column shows the number of covariates (MGS and clinical factors) used after removing near-zero-variance covariates. ARI cases (in percent) are computed in the training set and are used as the accuracy (ACC) reference that one can achieve by only predicting “yes” or “no” to all the samples. In-sample accuracy (ACC) refers to the model accuracy on the training set, while out-sample accuracy (ACC) refers to the model accuracy in the testing set. Sensitivity means the proportion of actual positive cases correctly identified, while specificity means the proportion of actual negative cases correctly identified. Download Table S3, DOCX file, 0.04 MB. Copyright © 2021 Binia et al. 2021 Binia et al. https://creativecommons.org/licenses/by/4.0/ This content is distributed under the terms of the Creative Commons Attribution 4.0 International license.

### Nasopharyngeal microbiome composition was associated with the presence of bacterial facultative pathogens.

The detection by culture and by MGS characterization of the three facultative pathogens (H. influenzae, S. pneumoniae, and M. catarrhalis) was highly consistent; 99% of samples in which a pathogen was detected by culture also showed the corresponding MGS (MGS abundance, >0) (Table S4). Further analysis was conducted to explore microbiome features specifically linked to the presence of pathogens.

10.1128/mSphere.00686-21.9 TABLE S4 Contingency table with sample counts showing concordance between the pathogen culture data and the MGS abundances. Download Table S4, DOCX file, 0.03 MB. Copyright © 2021 Binia et al. 2021 Binia et al. https://creativecommons.org/licenses/by/4.0/ This content is distributed under the terms of the Creative Commons Attribution 4.0 International license.

**FIG 6:**
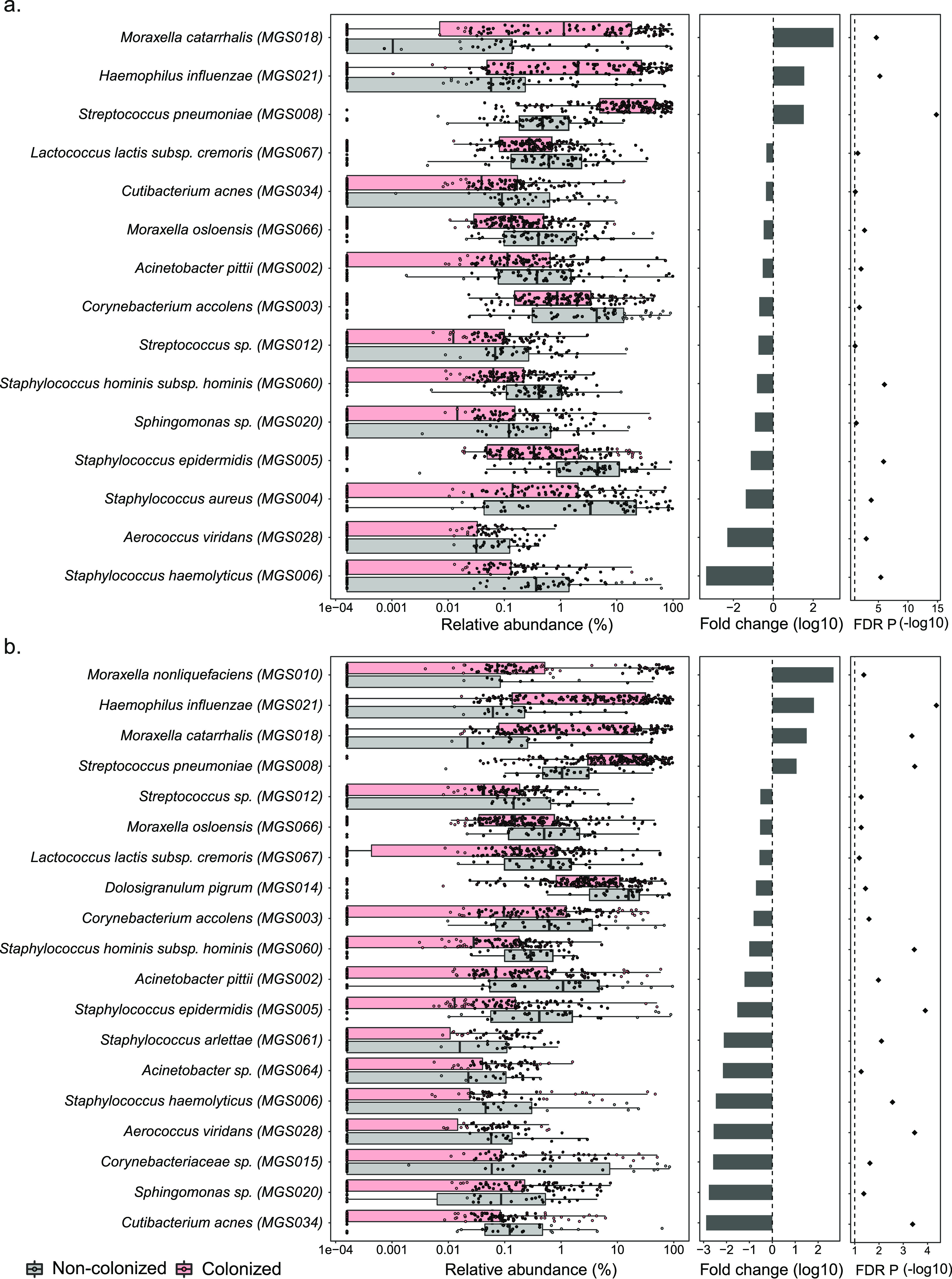
Differentially abundant metagenomic species between colonized and noncolonized samples (FDR *P < *0.1) at 2 months (a) and at 4 months (b). The left panels display the median and IQR of the relative abundance. For this figure, a small constant (minimum relative abundance observed divided by 2) was added to all abundances to avoid a logarithm of zero. The middle panels show the fold change, and the right panels show the FDR-adjusted *P* value (FDR P) of a two-sided Wilcoxon rank-sum test for the difference between colonized and noncolonized samples.

While no significant differences were observed in alpha-diversity between colonized (one of the three pathogens was detected by culture) and noncolonized samples, they strongly differed in microbiome composition. Beyond the expected differences in the 3 MGS matching the pathogens detected by culture (MGS008, MGS018, and MGS021), 12 MGS were significantly more abundant among the noncolonized samples at 2 months (two-sided Wilcoxon rank-sum test, FDR < 0.1) (Fig. 6), and this number increased to 16 by 4 months of age. All species showing higher abundance in noncolonized samples are considered to possess no or low pathogenic potential, except for S. aureus, which showed differential abundance only at 2 months and greatly decreased in overall abundance by 4 months of age. Moraxella nonliquefaciens was the only species more abundant in the colonized samples and only at 4 months, an effect seemingly driven by a subset of samples with a high abundance of this species (Figure 6b).

## DISCUSSION

### Maternal secretor and Lewis status as proxies of HMO composition in maternal milk and infant ARIs.

The Microbiota and Health Study provided an opportunity to assess a population of predominantly breastfed infants with high ARI prevalence, peaking between two and 6 months of age. We observed that positive maternal secretor status (defined by the presence of a functional variant of the *FUT2* gene) was associated with a reduced ARI risk. Remarkably, this effect was observed only during the first 6 months of life and thus coincided with the period of exclusive or predominant breastfeeding in the study population. Our study exploited a tight link between genetic variation in *FUT2* and *FUT3* determining secretor and Lewis status and the presence of a distinct group of 1,2- and 1,3/4-fucosylated HMOs in breast milk (10, 11). As maternal secretor status is a reliable marker of the availability of specific HMOs to a breastfed infant (11), our results suggest a protective role of 1,2-fucosylated HMOs against respiratory infections in early infancy. All mutations (in this case in *FUT2*) can have unknown pleiotropic effects; therefore, it cannot be excluded that the effects on the infants are conveyed by yet unknown mechanisms other the effect on HMO content of milk. For example, strong inhibition of Norwalk virus by secretor milk was conveyed not by free oligosaccharides but, rather, by glycoproteins (29). A mother’s secretor status could impact other aspects of her phenotype, including maternal microbiome, with a potential downstream effect on the infant.

Previous studies have suggested a role for 2′FL, which is the most abundant 1,2-fucosylated HMO in secretor milk, in protection against diarrhea in a cohort of Mexican infants (12, 13). However, no association between maternal secretor status and ARI risk was observed in a UK cohort (30). The infants in the latter study were only partially rather than exclusively breastfed, suggesting that exclusive breastfeeding may be necessary to observe a protective effect. Other variables, notably living environment, and ARI etiology could also explain the difference between the studies. Our observations suggesting the protective role of 1,2-fucosylated HMOs are consistent with the results of a randomized clinical trial that observed a reduced risk of LRTI and decreased need for antibiotics and antipyretics in young infants fed formula containing two HMOs, 2′FL and LNnT (15).

Other classes of HMOs might be involved in protection from respiratory infections, as suggested by a pilot study in the United States where the level of lacto-*N*-fucopentaose II (LNFP-II, an 1,3/4-fucosylated HMO) was associated with less respiratory illness in the first 4 months of life (31). In our study we did not identify any significant associations between ARI and maternal Lewis status, a proxy for 1,3/4-fucosylated HMO. However, our results cannot be directly compared to the above-mentioned study where only levels of LNFP-II in milk and no other HMOs were analyzed, in contrast to our approach using genetic markers as a proxy for groups of fucosylated HMO. Nevertheless, results from observational studies propose a protective role of fucosylated HMOs against respiratory infections that needs to be further understood.

Our study also examined the association between infant *FUT2* and *FUT3* polymorphisms and ARIs. Infant secretor but not Lewis status was weakly associated with increased ARI risk during the first 4 months of life. A large cohort study conducted in the United Kingdom found increased susceptibility to respiratory infections in secretor-positive infants occurring between 12 and 24 months, but not in early infancy (30). Earlier studies found that secretors of all ages combined were overrepresented among cases of respiratory infections and individuals diagnosed with respiratory viruses (32). While an individual’s secretor status seems to modulate the susceptibility to ARIs, a better understanding of its interaction with other factors such as age and etiology is needed.

### Protective effects of maternal secretor status were not conveyed by effects on respiratory pathogens.

We did not find substantial evidence that the protective effect of maternal HMOs is conveyed by modulation of respiratory pathogens. We observed a trend for reduced colonization of infants of secretor-positive mothers with bacterial facultative respiratory pathogens (S. pneumoniae, H. influenzae, M. catarrhalis), and the presence of any of these pathogens was weakly associated with ARI risk in the subsequent 2-month period. Variable results were reported on the link between colonization and risk of subsequent ARIs, in particular, contrasting findings from two studies evaluating the same set of pathogens conducted in relatively similar northern European populations (Denmark and the Netherlands) (21, 33). It appears that the association between pathogen colonization and later ARIs could be driven by more severe infections, LRTIs and bronchitis, where bacterial etiology is more usual, while no such link is observed for milder ARIs. We posit that bacterial pathogens are unlikely etiological ARI agents in our study due to the short duration of the majority of episodes (mean duration of 5 days; Vidal et al., in preparation) and human rhinovirus being a predominant etiological agent. The weak link between secretor-positive milk and reduced colonization on one hand and reduced colonization and subsequent infections on the other appears to more likely be an indicator of generalized immune support conveyed by feeding secretor-positive milk, rather than a direct effect of fucosylated HMO sequestering etiological agents of respiratory infections.

The high prevalence of human rhinovirus in asymptomatic infants (42% and 53% at 2 and 4 months, respectively) may appear surprising, suggesting a state of “colonization” with minimal symptoms and/or persistence of the virus after symptoms have ceased. These results agree with the findings of a large cohort study investigating the etiology of severe infections in South East Asia that also included healthy infants (34), where 34% of infants aged, on average, 13 days had human rhinovirus infection. The high colonization rate by facultative respiratory bacterial pathogens (S. pneumoniae, M. catarrhalis, H. influenzae) is in line with findings from other studies conducted in similar settings (35, 36). Despite variation among studies, higher prevalence appears to be observed in low-resource settings, e.g., 29% at 7 weeks of age in South Africa (37) compared to 8.5% at 6 weeks of age in the Netherlands (33).

Symptomatic infants appeared to be slightly but consistently more likely to be colonized by three main bacterial respiratory pathogens that were detected both by culture throughout the study period as well as by molecular methods at 2 and 4 months. In addition, K. pneumoniae (only detected by the molecular method) followed the same pattern. However, as bacterial etiology of ARIs appears unlikely, higher prevalence in symptomatic infants could be an indicator of compromised immunity.

### Protective effects of maternal secretor status were not conveyed by modulation of the nasopharyngeal microbiome.

Beyond respiratory pathogens, we also hypothesized that HMOs could shape the nasopharyngeal microbiome and thus modulate susceptibility to infections. To address this, the nasopharyngeal microbiome needs to be properly characterized up to the species level, notably to distinguish commensals from pathogens, which are often phylogenetically very close. Most previous studies described the nasopharyngeal microbiome in infancy using 16S rRNA gene sequencing (38–40), while shotgun metagenomics was only utilized in sick infants (41) or as a descriptive proof-of-concept study (42). To our knowledge this is the first study to examine the nasopharyngeal microbiome from a large cohort of healthy infants using shotgun metagenomics. A high-resolution profile of the microbiome composition up to at least the species level was achieved, despite the predominance of human sequence reads in some samples. The high level of consistency between shotgun metagenomics indicating the presence of H. influenzae, S. pneumoniae, or M. catarrhalis and culture data further confirmed the quality of the characterization.

Contrary to our hypothesis, we could not link the nasopharyngeal microbiome at 2 or 4 months in asymptomatic infants to subsequent ARI risk. A recent meta-analysis demonstrated consistent differences in respiratory microbiota between infants suffering from respiratory illnesses and healthy controls, manifesting mainly as increased presence of Haemophilus and Streptococcus and reduced diversity during the disease (D. Broderick, D. W. Waite, R. Marsh, C. A. Camargo, P. Cardenas, A. B. Chang, W. O. C. Cookson, L. Cuthbertson, W. Dai, M. L. Everard, A. Gervaix, J. K. Harris, K. Hasegawa, L. R. Hoffman, S. J. Hong, L. Josset, M. S. Kelly, B. S. Kim, K. Yong, S. C. Li, J. Mansbach, A. Mejias, G. A. O’toole, L. Paalanen, M. P. Losada, M. M. Pettigrew, M. Pichon, O. Ramilo, L. Ruokolainen, O. Sakwinska, P. Seed, C. J. Van Der Gast, B. Wagner, H. Yi, E. T. Zemanick, Y. Zheng, N. Pillarisetti, and M. Taylor, submitted for publication). Other studies showed some consistency in associating the lack of taxa such as *Dolosigranulum* and *Corynebacterium* with disease (43–45).

However, cross-sectional design cannot determine whether these changes were a cause or consequence of the disease. Few previous studies employed a longitudinal prospective design and suggested that specific microbiome profiles were associated with higher risk of subsequent ARIs (46–48); the results of these studies were not unequivocal. While an earlier study in the Netherlands reported that the respiratory microbiome dominated by *Moraxella* was associated with a lower incidence of subsequent respiratory infections (47), newer studies conducted in Finland and Australia showed that the *Moraxella*-dominated community type was associated with a higher incidence of ARIs (46, 48, 49). These contradictory findings could be a consequence of the lack of taxonomic resolution based on the sequencing of the 16S rRNA gene leading to variable classification within the *Moraxella* genus. While the pathogenic potential of M. catarrhalis is well established, this evidence has been emerging for *M. nonliquefaciens* through genome analysis (50) and associations with sinusitis (44) and viral pneumonia (39). In our data, *M. nonliquefaciens* showed a positive association with the presence of other established respiratory pathogens, while Moraxella osloensis displayed an inverse correlation with pathogen presence.

We documented striking antagonistic associations among a wide range of species and the presence of established respiratory pathogens, beyond the inverse association between S. pneumoniae and S. aureus carriage described before (51). Overall, these data could suggest competition for the same ecological niche. However, the host is not a passive element, and factors such as mucosal immunity likely shape the competitive balance among the nasopharyngeal microbiome community. Although our data did not lend support for the link between the nasopharyngeal microbiome and common uncomplicated ARIs, it is possible that more severe infections such as LRTIs or other respiratory conditions such as asthma later in life could be modulated by the microbiome composition (49). More targeted high-resolution methodology is needed to establish robust associations.

The prevailing environmental factor shaping the nasopharyngeal microbiome in our study was seasonality. Only a few previous studies have observed the same (38, 46). It is plausible that in our study population, the infants were more exposed to the external environment, in contrast to previous studies mostly conducted in industrialized settings. On the other hand, we did not observe the effect of several environmental drivers on the microbiome composition as reported before. Our study population was very homogenous in respect to breastfeeding and did not attend daycare; however, high exposure to other nonsibling children appeared very common, which together with the prevailing effect of the season could have stronger effects than other environmental factors.

### Alternative hypotheses.

We have not found compelling evidence that protective effects of HMOs were conveyed by modulation of the nasopharyngeal microbiome. This leads us to hypothesize that HMOs could exert an immunomodulatory effect on the host. As a nonexclusive alternative, HMOs shape the gut microbiome composition and function, leading to production of metabolites with an immunomodulatory effect on the host (“gut-lung axis”), and results from animal models lend some support to such a potential mechanism (52). Microbes residing in the gut are increasingly implicated in susceptibility to respiratory infections (53, 54). In the context of HMOs, a shift in the infant gut microbiome accompanied a reduction in respiratory infections observed in infants consuming two HMOs (2′FL and LNnT) (55).

### Limitations.

The results of genetic analyses should be interpreted with caution, as the study was of moderate size. The number of nonsecretor individuals was low, especially compared to previous studies in Bangladeshi populations (25, 56); however, this difference was not significant and was most likely due to limited sample size. Namely, in the study by Williams and colleagues (56) there were 20 nonsecretors and 94 secretors, and in ours, 24 nonsecretors and 186 secretors (Chi square test *P* = 0.125). In addition, no data on milk composition was available, and the extrapolation to HMO content relies on genetic markers. However, it is well established that major fucosylated HMOs such as 2′FL and LNFP-I or LNFP-II depend entirely on *FUT2* and *FUT3* genetic factors (10, 11). Still, even though genetic nonsecretors have virtually no fucosylated HMO in milk, the quantities vary substantially among individuals with intact *FUT2*. This typically leads to a higher proportion of nonsecretors when phenotypic criteria (antigen detection in saliva) are used (56) and could lead to the inability to detect true associations, in our case between infant outcomes and HMO content. Ideally, larger studies assessing both HMO milk content, genotyping of both maternal and infant *FUT2* and *FUT3*, and the gut and respiratory microbiome should be performed to provide further insights in the association among HMOs, genetic innate susceptibility, and infection risk.

The in-depth analysis of the nasopharyngeal microbiome and its associations with subsequent ARIs was focused on samples collected from asymptomatic infants at 2 and 4 months of age, when the peak prevalence of ARIs was observed in our study. However, detailed analysis revealed no predictive power regarding future ARI episodes. The presence of respiratory pathogens at birth appeared to be most associated with subsequent ARI episodes. It cannot be excluded that the very early microbiome before 2 months of age could provide predictive power regarding subsequent ARIs.

The etiology of the majority of ARIs could not be established, due to the lack of feasibility to collect samples during the episodes. However, the samples that were collected during scheduled visits provided a good snapshot of the prevailing etiology.

The information on ARI occurrence in our study was collected by active surveillance, but no symptom diaries or parental training was carried out. Our methodology was superior to many studies relying on parental recall only; nevertheless, some episodes were likely missed. Studies with a formal parental diary card listing predefined respiratory symptoms and parental training (57) reported higher incidence (e.g., 58). However, the potential underreporting should not be biased and therefore should not invalidate our conclusions.

### Conclusions.

In conclusion, our study provided further evidence for an association of fucosylated HMOs with reduced risk of respiratory infections in exclusively or predominantly breastfed infants. In our study, infant secretor status and colonization by respiratory pathogens were at best weak modulators of this risk, while the nasopharyngeal microbiome appeared to have no observed effect, suggesting that any protective effects of HMOs or inherent susceptibility are not conveyed via changes in the nasopharyngeal microbiome. Future larger studies are warranted to provide mechanistic insights on links between secretor status, HMOs, and risk of respiratory infections.

## MATERIALS AND METHODS

### Study population and sampling.

The Microbiota and Health Study was a prospective, community-based, longitudinal study of respiratory and gastrointestinal infections including 267 infants born in Nandipara, a peri-urban area near Dhaka, Bangladesh, between April 2013 and October 2016. The follow-up period started in the third trimester of the pregnancy and continued to 2 years of life, as previously described (24). Ethical approval was obtained from the local independent institutional review board. The trial was registered on Clinicaltrials.gov (registration no. NCT02361164). Written informed consent was obtained from all mothers before enrollment in the study.

For the present analysis, we have selected infants who were either partially or exclusively breastfed until at least 4 months of age, which constituted a large majority of infants in the cohort (Vidal et al., in preparation). An ARI episode was defined as one or more of the following symptoms: cough, runny nose, nasal congestion, ear discharge, and rapid breathing. A new ARI episode was defined as an episode starting after 7 symptom-free days from a previous episode. Incidence rate (IR) of ARIs in 100 infant-years (IY) was determined by dividing the total number of ARI episodes occurring among infants by the total number of days that each infant was followed (IY) multiplied by 100.

To determine *FUT2* and *FUT3* polymorphisms, saliva samples were collected from both mothers and infants using Oragene collection and DNA extraction kits (OG-500 and OG-575, respectively), which were subsequently used for human DNA extraction according to manufacturer recommendations.

Nasopharyngeal samples were collected from the infants at bi-monthly intervals during scheduled visits. The detection of three common facultative bacterial respiratory pathogens (Moraxella catarrhalis, Streptococcus pneumoniae, Haemophilus influenzae) was performed by culture using standard clinical diagnostics methods. The remaining part of the sample was frozen and stored for future molecular analysis of viral and bacterial respiratory pathogens and nasopharyngeal microbiome. Total nucleic acid extraction from nasopharyngeal samples was performed using the QIAamp MinElute virus spin kit (Qiagen, Valencia, CA) according to manufacturer instructions. The starting material was 200 μl of nasopharyngeal samples (NPS) spiked with 2.5E8 PFU of MS2 bacteriophage (ZeptoMetrix, Buffalo, NY). The resulting nucleic acid extract was subsequently used for the detection of respiratory pathogens with customized TaqMan array cards (TAC) and for the analysis of the nasopharyngeal microbiome using shotgun metagenomics.

All biological samples were initially stored at −20°C and subsequently transferred to −80°C until analysis.

### Genetic analyses of *FUT2* and *FUT3* from mothers and infants.

The entire *FUT2* and *FUT3* coding sequences and part of the 3′ and 5′ untranslated regions were PCR amplified for 30 cycles using the Kapa HiFi (Roche), starting from 50 ng DNA. The PCR primer sequences were ACACACCCACACTATGCCTG (FUT2-Fw), AAGAGAGATGGGTCCTGCTC (FUT2-Re), CCCGGAGCTTTGGTAAGCAG (FUT3-Fw), and GAGGGTTGGCCACAAAGGAC (FUT3-Re). The same melting temperature of 60°C was used for both amplifications. A positive control (DNA from HapMap NA18523) and a no-template control (water) were included on each PCR plate. The quality and quantity of each FUT2 and FUT3 PCR were checked by gel electrophoresis using the LabChip GX Touch (Perkin Elmer).

After purification on Ampure beads (Beckman) at a 1.8× ratio, sequencing libraries were prepared from the amplicons using the Nextera XT kit (Illumina) strictly following manufacturer’s recommendations. Libraries were quantified with Picogreen (Life Technologies), and their size pattern was validated with a Fragment Analyzer (AATI). Sequencing was performed as a paired-end 250-cycle run with the MiSeq Reagent kits v2 (Illumina).

Variant calling was performed with the software FreeBayes (v1.1.0-3-g961e5f3) (59) using default parameters. The resulting vcf files were postprocessed with the Plink software (v1.9) for quality control (QC) purpose and recoding. The sequence of QC steps was as follows: (i) samples with more than 5% of missing genotypes were filtered out; (ii) variants missing in more than 5% of the samples were filtered out; (iii) variants with a minor allele frequency (MAF) below 1% (computed on the cohort data set) were filtered out.

Individuals were defined as nonsecretors if they were homozygous for the minor allele of any of the following SNPs: rs601338, rs1047781, or rs200157007 (11, 25–27). Individuals were defined as Lewis negative if they were homozygous for the minor allele of any of the following SNPs: rs3745635, rs28362459, rs3894326, or rs812936 (11).

### Detection of respiratory pathogens by TaqMan array cards.

Nasopharyngeal samples collected at 2 and 4 months of age from infants asymptomatic for ARI were tested for 10 bacterial and 10 viral respiratory pathogens using customized TaqMan array cards (TAC) (Thermo Fisher Scientific, Waltham, MA) according to the methods outlined in Saha et al. 2018 (34). The design included one internal positive control (IPCO1) and two specimen quality control assays (RNaseP and MS2 bacteriophage). A total of 45 μl of nucleic acid extract was amplified with the qScript XLT 1-step RT-qPCR ToughMix Low-ROX kit (Quanta Biosciences, Gaithersburg, MD) for a total volume of 100 μl per testing lane. Six specimens were tested per card, along with a positive and negative control on each TAC. The negative control consisted of nuclease-free water, while the positive control was a pool of engineered templates that were designed to amplify at a consistent threshold cycle (*C_T_*) range (60). The following cycling conditions were used: 45°C for 10 min, 94°C for 10 min, and 45 cycles of 94°C for 30 s followed by 60°C for 1 min on a QuantStudio 7 real-time PCR system (Thermo Fisher Scientific). Data analysis was performed using the QuantStudio 7 software.

### Sequencing and analysis of the nasopharyngeal microbiome.

A total of 422 nasopharyngeal samples from 2- and 4-month-old infants asymptomatic for ARI at the time of sampling were available for shotgun metagenomics sequencing on the Illumina HiSeq platform (for details see Supplemental Text S1). Briefly, after quality control and host-read filtering, the remaining reads were *de novo* assembled using MEGAHIT, and genes were predicted on all samples using Prodigal. Gene predictions were merged with genes from respiratory tract microbial species sequenced by the Human Microbiome Project (61), and all genes were clustered at 95% similarity to build a nonredundant nasopharynx gene catalog. Gene abundances for each sample were then computed by mapping filtered reads to the gene catalog. Metagenomic species (MGS) were identified based on the coabundance of genes across the 422 samples, following the method defined of Nielsen and colleagues (62). To annotate the MGS, all the catalog genes were subjected to a BLAST search of the NCBI RefSeq genome database and used different levels of similarity (ranging from 65% for phylum to 95% for species) to annotate at the various taxonomic levels.

### Statistical analyses.

**Clinical data.** Statistical analyses of clinical data have been performed using SAS (v9.4). For computation of odds ratios (OR), incidence rate ratios (IRR), confidence intervals (CI) and *P* values, the logistic regression was used. The secretor and/or Lewis status of mothers and/or infants were included as covariates. When the response was measured repeatedly, the repeated statement for subject and the covariate “visit” were included in the models as well. IRR were used to compare the number of ARIs in given time periods between groups, whereas OR compared the occurrence of at least one ARI between groups. The Wald test was used to compare proportions of infants colonized by respiratory pathogens between groups symptomatic and asymptomatic for ARI at the time of sample collection.

**Methods used to associate colonization by respiratory pathogens with ARI.** The association between respiratory pathogen colonization at 2 and 4 months and subsequent ARI was evaluated through a Kaplan-Meier analysis, including only pathogens for which at least 20 subjects tested positive.

**Machine learning approaches to associate microbiome with explanatory variables and risk of ARI.** Machine learning analyses on microbiome composition are detailed in Supplemental Text S1. Briefly, the caret package v6.0-80 (63) in R (v3.5.0) was used to predict whether there was an ARI over the next 1, 2, 6, or 20 months after sampling based on microbiome composition at 2 or 4 months together with selected clinical factors. Models were built using three different approaches—GLMnet, random forest, and LogitBoost—and microbiome abundance data were transformed as either (i) presence/absence (0/1), (ii) raw abundance percentages, or (iii) log_10_-transformed. Samples were partitioned into training and testing sets at a ratio of 80/20%, and results were evaluated considering accuracy, sensitivity, and specificity.

### Data availability.

Genetic data and raw nasopharyngeal microbiome reads depleted of human sequences were deposited in the European Nucleotide Archive (ENA) under the project accession number PRJEB42539 including descriptive metadata. Additional metadata can be shared by the corresponding author on reasonable request.
