# Supplementary material for: The Influence of FUT2 and FUT3 Polymorphisms and Nasopharyngeal Microbiome on Respiratory Infections in Breastfed Bangladeshi Infants from the Microbiota and Health Study
Source: mSphere. 2021 Nov 10;6(6):e00686-21. doi: 10.1128/mSphere.00686-21 (PMC8579893; doi:10.1128/mSphere.00686-21)
Supplement: TABLE S1 [file msphere.00686-21-st001.docx]

| **SNP** | **Gene** | **Chromosome** | **Position** | **MAF** | **Group** |
| --- | --- | --- | --- | --- | --- |
| rs150146742 | FUT3 | 19 | 5843740 | 0.01584 | Mother |
| rs3894326 | FUT3 | 19 | 5843773 | 0.1357 | Mother |
| rs148170391 | FUT3 | 19 | 5844195 | 0.01357 | Mother |
| rs28362465 | FUT3 | 19 | 5844228 | 0.01131 | Mother |
| rs3745635 | FUT3 | 19 | 5844332 | 0.06561 | Mother |
| rs778986 | FUT3 | 19 | 5844526 | 0.1222 | Mother |
| rs812936 | FUT3 | 19 | 5844638 | 0.1312 | Mother |
| rs28362459 | FUT3 | 19 | 5844781 | 0.2036 | Mother |
| rs516316 | FUT2 | 19 | 48702888 | 0.2308 | Mother |
| rs516246 | FUT2 | 19 | 48702915 | 0.2285 | Mother |
| rs492602 | FUT2 | 19 | 48703160 | 0.2308 | Mother |
| rs681343 | FUT2 | 19 | 48703205 | 0.2308 | Mother |
| rs149356814 | FUT2 | 19 | 48703267 | 0.01131 | Mother |
| rs200157007 | FUT2 | 19 | 48703291 | 0.2014 | Mother |
| rs281377 | FUT2 | 19 | 48703346 | 0.4932 | Mother |
| rs1047781 | FUT2 | 19 | 48703374 | 0.07014 | Mother |
| rs601338 | FUT2 | 19 | 48703417 | 0.2308 | Mother |
| rs1800027 | FUT2 | 19 | 48703469 | 0.0543 | Mother |
| rs602662 | FUT2 | 19 | 48703728 | 0.2308 | Mother |
| rs485186 | FUT2 | 19 | 48703949 | 0.2308 | Mother |
| rs485073 | FUT2 | 19 | 48703998 | 0.2308 | Mother |
| rs603985 | FUT2 | 19 | 48704000 | 0.2308 | Mother |
| rs150146742 | FUT3 | 19 | 5843740 | 0.02283 | Infant |
| rs3894326 | FUT3 | 19 | 5843773 | 0.1256 | Infant |
| rs148170391 | FUT3 | 19 | 5844195 | 0.01142 | Infant |
| rs28362465 | FUT3 | 19 | 5844228 | 0.01142 | Infant |
| rs3745635 | FUT3 | 19 | 5844332 | 0.07534 | Infant |
| rs778986 | FUT3 | 19 | 5844526 | 0.1005 | Infant |
| rs812936 | FUT3 | 19 | 5844638 | 0.1073 | Infant |
| rs28362459 | FUT3 | 19 | 5844781 | 0.2032 | Infant |
| rs145362171 | FUT3 | 19 | 5844793 | 0.01142 | Infant |
| rs516316 | FUT2 | 19 | 48702888 | 0.2283 | Infant |
| rs516246 | FUT2 | 19 | 48702915 | 0.2283 | Infant |
| rs492602 | FUT2 | 19 | 48703160 | 0.2283 | Infant |
| rs681343 | FUT2 | 19 | 48703205 | 0.2283 | Infant |
| rs149356814 | FUT2 | 19 | 48703267 | 0.0274 | Infant |
| rs200157007 | FUT2 | 19 | 48703291 | 0.1872 | Infant |
| rs281377 | FUT2 | 19 | 48703346 | 0.4886 | Infant |
| rs1047781 | FUT2 | 19 | 48703374 | 0.07763 | Infant |
| rs601338 | FUT2 | 19 | 48703417 | 0.2283 | Infant |
| rs1800027 | FUT2 | 19 | 48703469 | 0.07078 | Infant |
| rs602662 | FUT2 | 19 | 48703728 | 0.2283 | Infant |
| rs144269088 | FUT2 | 19 | 48703857 | 0.01142 | Infant |
| rs485186 | FUT2 | 19 | 48703949 | 0.2283 | Infant |
| rs485073 | FUT2 | 19 | 48703998 | 0.2283 | Infant |
| rs603985 | FUT2 | 19 | 48704000 | 0.2283 | Infant |
