# Supplementary material for: The Influence of FUT2 and FUT3 Polymorphisms and Nasopharyngeal Microbiome on Respiratory Infections in Breastfed Bangladeshi Infants from the Microbiota and Health Study
Source: mSphere. 2021 Nov 10;6(6):e00686-21. doi: 10.1128/mSphere.00686-21 (PMC8579893; doi:10.1128/mSphere.00686-21)
Supplement: TABLE S2 [file msphere.00686-21-st002.docx]

| **Variable** | **Category** | **IRR (95% CI)** | ***P*** |
| --- | --- | --- | --- |
| **Any pathogen** |  |  |  |
| Age | Increment by 30 days | 1.19 (1.05 to 1.34) | 0.0048 |
| ARI in the first 2 months | 0: No |  |  |
|  | 1: Yes | 2.77 (2.05 to 3.73) | <.0001 |
| Breastfeeding status | 0: EB |  |  |
|  | 1: PB | 1.30 (0.39 to 4.26) | 0.6698 |
| Colonization status | 0: No |  |  |
|  | 1: Yes | 1.59 (1.01 to 2.53) | 0.0470 |
| Infant Lewis type | 0: Le- |  |  |
|  | 1: Le+ | 0.90 (0.48 to 1.69) | 0.7427 |
| Infant Secretor type | 0: Se- |  |  |
|  | 1: Se+ | 1.29 (0.86 to 1.96) | 0.2227 |
| Maternal Lewis type | 0: Le- |  |  |
|  | 1: Le+ | 1.37 (0.85 to 2.22) | 0.1933 |
| Maternal Secretor type | 0: Se- |  |  |
|  | 1: Se+ | 0.85 (0.54 to 1.33) | 0.4802 |
| Season at episode | 1. Pre-monsoon (dry) hot (MAR-MAY) |  |  |
|  | 2. Rainy monsoon (JUN-OCT) | 1.78 (1.16 to 2.72) | 0.0082 |
|  | 3. Cool dry winter (NOV-FEB) | 1.00 (0.59 to 1.70) | 0.9936 |
| ***Haemophilus influenzae*** |  |  |  |
| Age | Increment by 30 days | 1.27 (1.16 to 1.40) | <.0001 |
| ARI in the first 2 months | 0: No |  |  |
|  | 1: Yes | 2.73 (2.00 to 3.71) | <.0001 |
| Breastfeeding status | 0: EB |  |  |
|  | 1: PB | 1.33 (0.43 to 4.12) | 0.6240 |
| Colonization status | 0: No |  |  |
|  | 1: Yes | 1.11 (0.75 to 1.65) | 0.5931 |
| Infant Lewis type | 0: Le- |  |  |
|  | 1: Le+ | 0.97 (0.50 to 1.87) | 0.9282 |
| Infant Secretor type | 0: Se- |  |  |
|  | 1: Se+ | 1.27 (0.83 to 1.95) | 0.2727 |
| Maternal Lewis type | 0: Le- |  |  |
|  | 1: Le+ | 1.48 (0.92 to 2.36) | 0.1037 |
| Maternal Secretor type | 0: Se- |  |  |
|  | 1: Se+ | 0.87 (0.56 to 1.36) | 0.5422 |
| Season at episode | 1. Pre-monsoon (dry) hot (MAR-MAY) |  |  |
|  | 2. Rainy monsoon (JUN-OCT) | 1.72 (1.12 to 2.65) | 0.0137 |
|  | 3. Cool dry winter (NOV-FEB) | 1.03 (0.60 to 1.77) | 0.9232 |
| ***Moraxella catarrhalis*** |  |  |  |
| Age | Increment by 30 days | 1.24 (1.13 to 1.35) | <.0001 |
| ARI in the first 2 months | 0: No |  |  |
|  | 1: Yes | 2.67 (1.95 to 3.66) | <.0001 |
| Breastfeeding status | 0: EB |  |  |
|  | 1: PB | 1.35 (0.42 to 4.33) | 0.6164 |
| Colonization status | 0: No |  |  |
|  | 1: Yes | 1.64 (1.12 to 2.40) | 0.0113 |
| Infant Lewis type | 0: Le- |  |  |
|  | 1: Le+ | 0.98 (0.49 to 1.92) | 0.9430 |
| Infant Secretor type | 0: Se- |  |  |
|  | 1: Se+ | 1.27 (0.83 to 1.95) | 0.2643 |
| Maternal Lewis type | 0: Le- |  |  |
|  | 1: Le+ | 1.56 (0.97 to 2.52) | 0.0657 |
| Maternal Secretor type | 0: Se- |  |  |
|  | 1: Se+ | 0.90 (0.56 to 1.42) | 0.6424 |
| Season at episode | 1. Pre-monsoon (dry) hot (MAR-MAY) |  |  |
|  | 2. Rainy monsoon (JUN-OCT) | 1.87 (1.23 to 2.85) | 0.0033 |
|  | 3. Cool dry winter (NOV-FEB) | 0.94 (0.55 to 1.62) | 0.8285 |
| ***Streptococcus pneumoniae*** |  |  |  |
| Age | Increment by 30 days | 1.25 (1.13 to 1.39) | <.0001 |
| ARI in the first 2 months | 0: No |  |  |
|  | 1: Yes | 2.75 (2.02 to 3.75) | <.0001 |
| Breastfeeding status | 0: EB |  |  |
|  | 1: PB | 1.30 (0.41 to 4.16) | 0.6586 |
| Colonization status | 0: No |  |  |
|  | 1: Yes | 1.22 (0.80 to 1.87) | 0.3642 |
| Infant Lewis type | 0: Le- |  |  |
|  | 1: Le+ | 0.93 (0.48 to 1.82) | 0.8344 |
| Infant Secretor type | 0: Se- |  |  |
|  | 1: Se+ | 1.30 (0.85 to 2.00) | 0.2242 |
| Maternal Lewis type | 0: Le- |  |  |
|  | 1: Le+ | 1.43 (0.89 to 2.30) | 0.1418 |
| Maternal Secretor type | 0: Se- |  |  |
|  | 1: Se+ | 0.85 (0.54 to 1.33) | 0.4747 |
| Season at episode | 1. Pre-monsoon (dry) hot (MAR-MAY) |  |  |
|  | 2. Rainy monsoon (JUN-OCT) | 1.75 (1.13 to 2.70) | 0.0120 |
|  | 3. Cool dry winter (NOV-FEB) | 1.03 (0.60 to 1.79) | 0.9089 |
