# Supplementary figures and images for: The Influence of FUT2 and FUT3 Polymorphisms and Nasopharyngeal Microbiome on Respiratory Infections in Breastfed Bangladeshi Infants from the Microbiota and Health Study
Source: mSphere. 2021 Nov 10;6(6):e00686-21. doi: 10.1128/mSphere.00686-21 (PMC8579893; doi:10.1128/mSphere.00686-21)

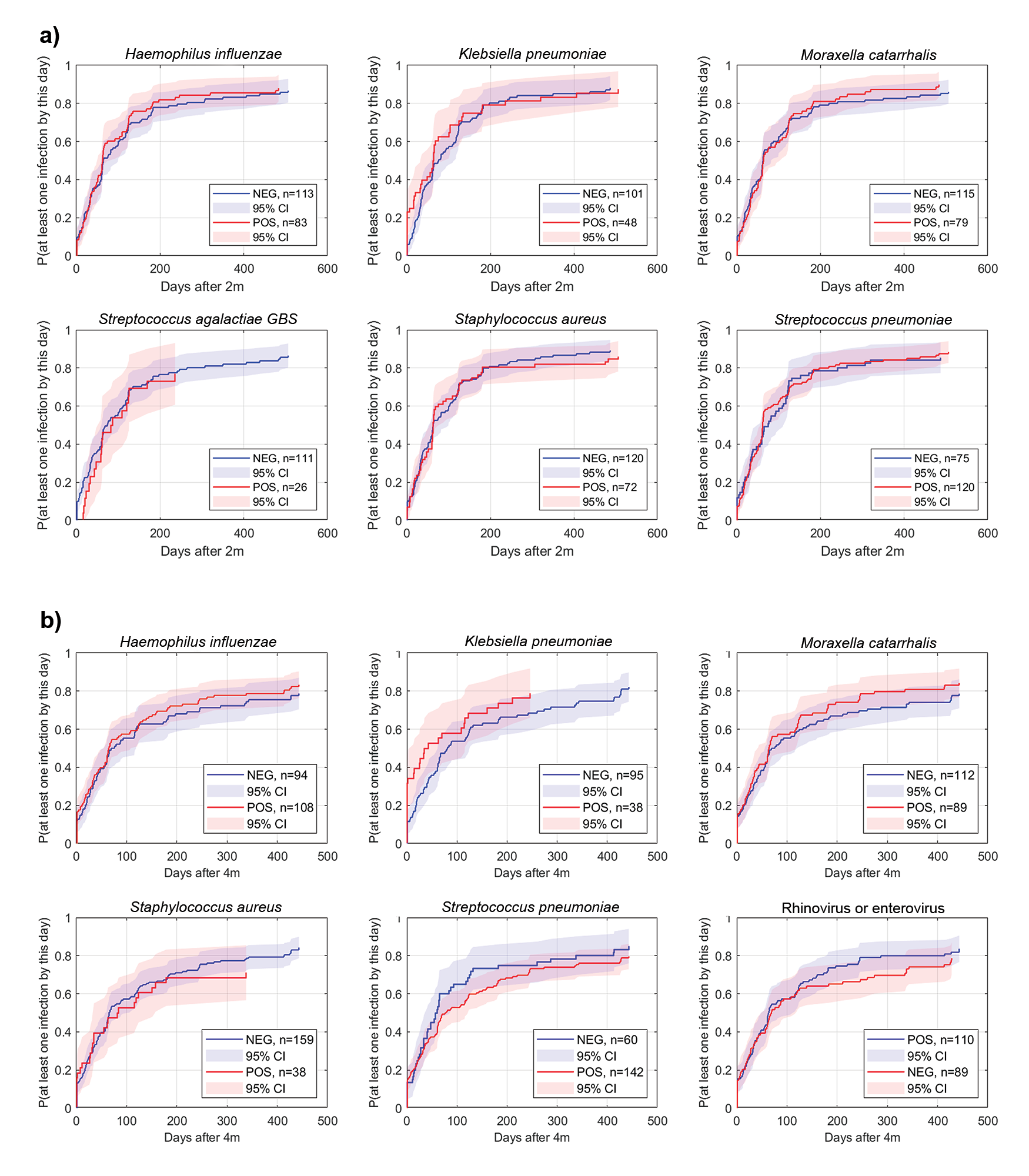

Supplement: FIG S2 [file msphere.00686-21-sf002.docx]

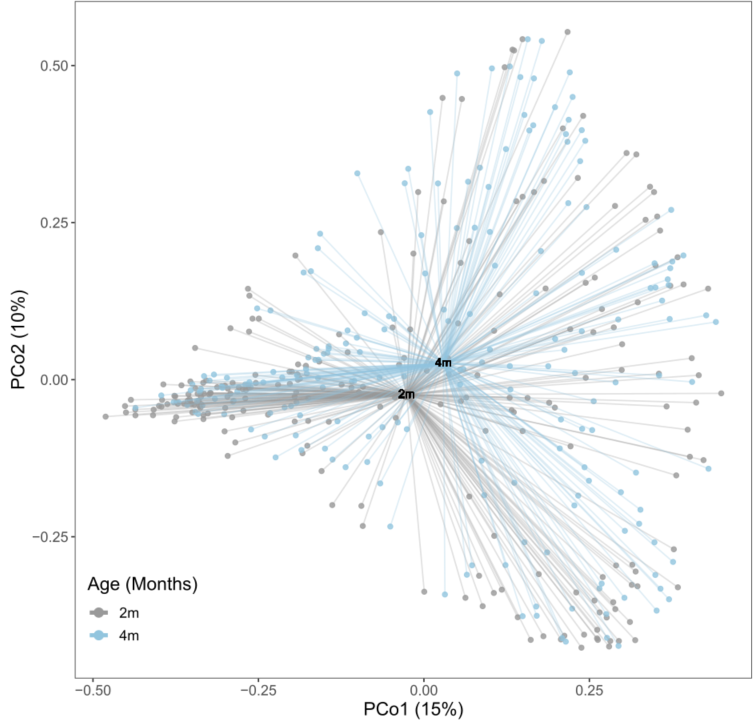

Supplement: FIG S3 [file msphere.00686-21-sf003.docx]
