# Supplementary material for: The Influence of FUT2 and FUT3 Polymorphisms and Nasopharyngeal Microbiome on Respiratory Infections in Breastfed Bangladeshi Infants from the Microbiota and Health Study
Source: mSphere. 2021 Nov 10;6(6):e00686-21. doi: 10.1128/mSphere.00686-21 (PMC8579893; doi:10.1128/mSphere.00686-21)
Supplement: TEXT S1 [file msphere.00686-21-s0001.docx]

# **Supplementary Methods****: Nasopharyngeal microbiome sequencing & data analysis**

*Microbiome sequencing*

Genomic DNA was randomly sheared into fragments of approximately 350 bp. The fragmented DNA was used for library construction using NEBNext Ultra II Library Prep Kit for Illumina (New England Biolabs). The library was sequenced using 2 x 150 bp paired-end sequencing on an Illumina HiSeq platform, at an average depth of 11.4 gigabases (Gb) (Min = 6.0 Gb and Max = 28.0 Gb) per sample, and with a minimum of 20 million (M) read pairs per sample.

*Preprocessing the sequencing data*

Sequencing data was quality controlled using Trim Galore v0.4.2^1^ and PRINSEQ v0.20.4^2^ to remove Illumina adapters, low complexity reads and duplicate reads, which could reduce assembly quality when generating the gene catalog. Reads were also trimmed using Trimmomatic v0.36^3^ by cutting the first 10 bp of the read (higher error rate due to unspecific hex-primers binding), requiring a minimum average Phred quality of 20 over a sliding window of 4 bp from the 5’ end of the read and cutting bases from the 3’ end if the quality dropped below 20. Furthermore, reads mapping to the human reference genome (GRCh38) were removed using Bowtie v2.3.2^4^. Finally, reads shorter than 100 bp were removed. Remaining reads were considered high quality reads; there was on average 4 M high quality host-filtered read pairs per sample (Min = 0.06 M and Max = 27.5 M).

*Building the nasopharyngeal gene catalog*

HQ reads were assembled into contigs of at least 500 bp using MEGAHIT v1.1.1^5^, and genes were predicted using Prodigal v2.6.2^6^. Gene predictions from the 422 nasopharyngeal samples were merged with genes from respiratory tract microbial species sequenced by the Human Microbiome Project^7^ (635 microbial assemblies from nasopharyngeal areas). A non-redundant nasopharyngeal catalog with 3,110,772 genes was then built by clustering the genes at minimum of 95 % sequence identity and requiring 90 % coverage of the shorter sequence and a gene length of at least 102 nucleotides (CD-HIT v4.6.1^8^). High quality reads were mapped to the catalog genes using BWA v0.7.16a^9^ for the alignments and SAMtools v1.6^10^ for merging. Repetitive sequences were identified by screening the gene catalog genes for interspersed repeats and low complexity DNA using RepeatMasker v4.0.7e^11^ (requiring a match of at least 75 bp and at least 75 % similarity) using a human repetitive elements database provided in the RepeatMasker package and a custom made nasopharyngeal-repeat database built with RepeatScout v1.0.5^12^ based on the gene catalog sequences. Repetitive sequences matching these criteria were considered low-quality signal and removed from the abundance calculations.

*Defining and profiling of metagenomics species*

Using the nasopharyngeal gene catalog, metagenomics species (MGSs) were identified based on the co-abundance of genes across the 422 infant stool samples. Clusters of genes that passed quality assessment (> 700 genes with inter-gene Pearson correlation coefficient (PCC) > 0.9) were defined as MGSs (method further explained in HB Nielsen *et al.*^13^) and expanded to 0.5 PCC for genes with consistent genus or species level taxonomy and base composition. For each MGS, the core genes set was defined as the 100 best abundance representative genes across the 422 samples. To calculate relative abundances of the MGSs, high-quality non-human sequencing reads were mapped to the nasopharyngeal gene catalog, requiring 95% identity in an alignment of at least 100 bases, and rejecting an alignment if >10 bases misaligned at either read end. Ambiguously mapped reads were ignored. Based on these mappings, length-normalized gene depths (reads/bp) were calculated for all genes. Then, each MGS depth was calculated as the average gene depth across its 100 core genes. The MGS depths were then scaled relative to the total number of high-quality non-host sequences reads, including the reads that did not map to the catalog genes (scaling factor = all MGS mapped reads / all reads). Furthermore, a MGS was considered detected only if high-quality reads were mapped to at least three of its 100 core genes. MGSs that did not satisfy this criterion were set to an abundance of zero. To annotate the MGSs, all the catalog genes were blasted to the NCBI RefSeq genome database and used different levels of similarity (ranging from 65 % for phylum to 95 % for species) to annotate at the various taxonomic level, all requiring a minimum of 80 % sequence coverage. Species level taxonomy was assigned to an MGS if > 75 % of its genes could be annotated to a given species. For genus, family, order, class and phylum, this percentage was adjusted to 60 %, 50 %, 40 %, 30 % and 25 % consistency respectively. Furthermore, at species and genus level, a maximum of 10 % of the genes in an MGS was allowed to be annotated to an alternative taxon. (i.e. up to 25% of the genes were allowed not to align to references from the primary taxon, as long as at most 10% of those genes aligned to alternative taxa). 3 MGSs were removed from the analyses and from the relative abundance calculations: MGS063 and MGS040 were identified as non-cellular organisms; MGS001, annotated as *Escherichia coli*, was considered contamination likely originating from a positive control spike-in before the DNA extraction.

*Machine learning approaches to associate microbiome with explanatory variables and risk of respiratory infections*

Machine learning analyses were performed using the caret package v6.0-80^14^ in R v3.5.0^15^, using microbiome composition at 2 and 4 months and clinical factors to predict whether there was an ARI 1, 2, 6 months after sampling up to the end of the study (i.e. 22 months after 2 months sampling and 20 months after 4 months sampling).

Clinical factors included were:

- Infant sex
- Mode of delivery
- Maternal Secretor status
- Infant Secretor status
- Infant birth weight
- Season of delivery
- Breastfeeding at 2 or 4 months
- Child colonization at 2 or 4 months
- Mother colonization at 4 months

Models were built using three different approaches: GLMnet, Random forests and LogitBoost. For all three approaches, parameters were tuned to improve the model performance (lambda and alpha for GLMnet, number of trees used for Random forests and number of iterations for LogitBoost). Preprocessing of the data included removing data with unknowns (“NA”) and near zero variance predictors. The abundance data was transformed as i) presence / absence (0/1) ii) raw abundance percentages or iii) log10-transformed abundance data. Samples were partitioned into training and testing sets at a ratio of 80/20%. Models were run on the training set using 10-fold cross-validation with 10 repetitions to optimize the models by assessing the best value for the algorithm parameters. For that, data was split in 10 separate groups; the models were trained on 9 sets and tested on the remaining set. This procedure was repeated 10 times and the performance results were averaged over these 10 experiments to reduce variability. Once the model was optimized, its performance was assessed on the testing set by analyzing:

i) accuracy ((TP+TN)/(TP+TN+FP+FN)),

ii) sensitivity (TP/TP+FN),

iii) specificity (TN/TN+FP),

where TP = True positive; FP = False positive; TN = True negative; FN = False negative.

*References*

1. Krueger F. Trim galore. *A wrapper tool around Cutadapt and FastQC to consistently apply quality and adapter trimming to FastQ files.* 2015.

2. Schmieder R, Edwards R. Quality control and preprocessing of metagenomic datasets. *Bioinformatics.* 2011;27(6):863-864.

3. Bolger AM, Lohse M, Usadel B. Trimmomatic: a flexible trimmer for Illumina sequence data. *Bioinformatics.* 2014;30(15):2114-2120.

4. Langmead B, Trapnell C, Pop M, Salzberg SL. Ultrafast and memory-efficient alignment of short DNA sequences to the human genome. *Genome Biol.* 2009;10(3):R25.

5. Li D, Luo R, Liu CM, et al. MEGAHIT v1.0: A fast and scalable metagenome assembler driven by advanced methodologies and community practices. *Methods.* 2016;102:3-11.

6. Hyatt D, Chen GL, Locascio PF, Land ML, Larimer FW, Hauser LJ. Prodigal: prokaryotic gene recognition and translation initiation site identification. *BMC Bioinformatics.* 2010;11:119.

7. Human Microbiome Project C. A framework for human microbiome research. *Nature.* 2012;486(7402):215-221.

8. Fu L, Niu B, Zhu Z, Wu S, Li W. CD-HIT: accelerated for clustering the next-generation sequencing data. *Bioinformatics.* 2012;28(23):3150-3152.

9. Li H, Durbin R. Fast and accurate long-read alignment with Burrows-Wheeler transform. *Bioinformatics.* 2010;26(5):589-595.

10. Li H, Handsaker B, Wysoker A, et al. The Sequence Alignment/Map format and SAMtools. *Bioinformatics.* 2009;25(16):2078-2079.

11. Smit A, Hubley R, Green P. RepeatMasker Open-4.0. 2013–2015. In:2015.

12. Price AL, Jones NC, Pevzner PA. De novo identification of repeat families in large genomes. *Bioinformatics.* 2005;21 Suppl 1:i351-358.

13. Nielsen HB, Almeida M, Juncker AS, et al. Identification and assembly of genomes and genetic elements in complex metagenomic samples without using reference genomes. *Nature biotechnology.* 2014;32(8):822-828.

14. Kuhn M. Building predictive models in R using the caret package. *Journal of statistical software.* 2008;28(5):1-26.

15. Team RC. R: A language and environment for statistical computing. In: Vienna, Austria; 2013.
