# Supplementary material for: The Influence of FUT2 and FUT3 Polymorphisms and Nasopharyngeal Microbiome on Respiratory Infections in Breastfed Bangladeshi Infants from the Microbiota and Health Study
Source: mSphere. 2021 Nov 10;6(6):e00686-21. doi: 10.1128/mSphere.00686-21 (PMC8579893; doi:10.1128/mSphere.00686-21)
Supplement: TABLE S3 [file msphere.00686-21-st003.docx]

**a) From 2 months**

| **Method** | **Period (months)** | **Abund data** | **N** | **Cov** | **ARI cases (%)** | **In-sample ACC (%)** | **Out-sample ACC (%)** | | **Sensitivity (%)** | **Specificity (%)** |
| --- | --- | --- | --- | --- | --- | --- | --- | --- | --- | --- |
| Glmnet | 2-3 | 0/1 | 208 | 48 | 17 | 88 | 65 | 14 | | 76 |
| RF | 2-3 | 0/1 | 208 | 48 | 17 | 100 | 83 | 0 | | 100 |
| LogitBoost | 2-3 | 0/1 | 208 | 48 | 17 | 91 | 76 | 14 | | 88 |
| Glmnet | 2-4 | 0/1 | 208 | 48 | 39 | 68 | 51 | 6 | | 80 |
| RF | 2-4 | 0/1 | 208 | 48 | 39 | 100 | 54 | 0 | | 88 |
| LogitBoost | 2-4 | 0/1 | 208 | 48 | 39 | 72 | 51 | 38 | | 60 |
| Glmnet | 2-8 | 0/1 | 207 | 48 | 73 | 82 | 63 | 83 | | 9 |
| RF | 2-8 | 0/1 | 207 | 48 | 73 | 100 | 73 | 100 | | 0 |
| LogitBoost | 2-8 | 0/1 | 207 | 48 | 73 | 73 | 61 | 80 | | 9 |
| Glmnet | 2-24 | 0/1 | 186 | 49 | 86 | 93 | 75 | 84 | | 20 |
| RF | 2-24 | 0/1 | 186 | 49 | 86 | 100 | 86 | 100 | | 0 |
| LogitBoost | 2-24 | 0/1 | 186 | 49 | 86 | 87 | 86 | 97 | | 20 |
| Glmnet | 2-3 | % | 208 | 45 | 17 | 83 | 80 | 0 | | 97 |
| RF | 2-3 | % | 208 | 45 | 17 | 100 | 83 | 0 | | 100 |
| LogitBoost | 2-3 | % | 208 | 45 | 17 | 98 | 66 | 43 | | 70 |
| Glmnet | 2-4 | % | 208 | 45 | 39 | 78 | 49 | 38 | | 56 |
| RF | 2-4 | % | 208 | 45 | 39 | 82 | 61 | 56 | | 64 |
| LogitBoost | 2-4 | % | 208 | 45 | 39 | 89 | 51 | 56 | | 48 |
| Glmnet | 2-8 | % | 207 | 45 | 73 | 81 | 61 | 67 | | 45 |
| RF | 2-8 | % | 207 | 45 | 73 | 100 | 71 | 97 | | 0 |
| LogitBoost | 2-8 | % | 207 | 45 | 73 | 94 | 61 | 70 | | 36 |
| Glmnet | 2-24 | % | 186 | 45 | 86 | 89 | 83 | 97 | | 0 |
| RF | 2-24 | % | 186 | 45 | 86 | 100 | 86 | 100 | | 0 |
| LogitBoost | 2-24 | % | 186 | 45 | 86 | 93 | 78 | 90 | | 0 |
| Glmnet | 2-3 | Log10 | 208 | 45 | 17 | 87 | 68 | 0 | | 82 |
| RF | 2-3 | Log10 | 208 | 45 | 17 | 100 | 85 | 14 | | 100 |
| LogitBoost | 2-3 | Log10 | 208 | 45 | 17 | 98 | 66 | 43 | | 70 |
| Glmnet | 2-4 | Log10 | 208 | 45 | 39 | 66 | 61 | 25 | | 84 |
| RF | 2-4 | Log10 | 208 | 45 | 39 | 79 | 49 | 44 | | 52 |
| LogitBoost | 2-4 | Log10 | 208 | 45 | 39 | 89 | 51 | 56 | | 48 |
| Glmnet | 2-8 | Log10 | 207 | 45 | 73 | 74 | 73 | 97 | | 9 |
| RF | 2-8 | Log10 | 207 | 45 | 73 | 100 | 71 | 93 | | 9 |
| LogitBoost | 2-8 | Log10 | 207 | 45 | 73 | 94 | 61 | 70 | | 36 |
| Glmnet | 2-24 | Log10 | 186 | 45 | 86 | 92 | 72 | 84 | | 0 |
| RF | 2-24 | Log10 | 186 | 45 | 86 | 100 | 86 | 100 | | 0 |
| LogitBoost | 2-24 | Log10 | 186 | 45 | 86 | 93 | 78 | 90 | | 0 |

**b) From 4 months**

| **Method** | **Period (months)** | **Abund data** | **N** | **Cov** | **ARI cases (%)** | **In-sample ACC (%)** | **Out-sample ACC (%)** | | **Sensitivity (%)** | **Specificity (%)** |
| --- | --- | --- | --- | --- | --- | --- | --- | --- | --- | --- |
| Glmnet | 4-5 | 0/1 | 198 | 51 | 16 | 82 | 79 | 0 | | 94 |
| RF | 4-5 | 0/1 | 198 | 51 | 16 | 100 | 84 | 0 | | 100 |
| LogitBoost | 4-5 | 0/1 | 198 | 51 | 16 | 86 | 71 | 17 | | 81 |
| Glmnet | 4-6 | 0/1 | 198 | 51 | 36 | 77 | 53 | 21 | | 72 |
| RF | 4-6 | 0/1 | 198 | 51 | 36 | 100 | 62 | 14 | | 88 |
| LogitBoost | 4-6 | 0/1 | 198 | 51 | 36 | 79 | 54 | 36 | | 64 |
| Glmnet | 4-10 | 0/1 | 197 | 50 | 62 | 78 | 69 | 75 | | 60 |
| RF | 4-10 | 0/1 | 197 | 50 | 62 | 100 | 69 | 96 | | 27 |
| LogitBoost | 4-10 | 0/1 | 197 | 50 | 62 | 75 | 67 | 79 | | 47 |
| Glmnet | 4-24 | 0/1 | 178 | 51 | 77 | 90 | 60 | 78 | | 0 |
| RF | 4-24 | 0/1 | 178 | 51 | 77 | 100 | 74 | 93 | | 12 |
| LogitBoost | 4-24 | 0/1 | 178 | 51 | 77 | 91 | 57 | 63 | | 38 |
| Glmnet | 4-5 | % | 198 | 48 | 16 | 86 | 84 | 17 | | 97 |
| RF | 4-5 | % | 198 | 48 | 16 | 100 | 84 | 0 | | 100 |
| LogitBoost | 4-5 | % | 198 | 48 | 16 | 92 | 74 | 17 | | 84 |
| Glmnet | 4-6 | % | 198 | 48 | 36 | 74 | 64 | 21 | | 88 |
| RF | 4-6 | % | 198 | 48 | 36 | 100 | 59 | 0 | | 92 |
| LogitBoost | 4-6 | % | 198 | 48 | 36 | 89 | 54 | 50 | | 56 |
| Glmnet | 4-10 | % | 197 | 48 | 62 | 75 | 46 | 54 | | 33 |
| RF | 4-10 | % | 197 | 48 | 62 | 100 | 67 | 92 | | 26 |
| LogitBoost | 4-10 | % | 197 | 48 | 62 | 97 | 67 | 83 | | 40 |
| Glmnet | 4-24 | % | 178 | 48 | 77 | 85 | 74 | 96 | | 0 |
| RF | 4-24 | % | 178 | 48 | 77 | 100 | 74 | 96 | | 0 |
| LogitBoost | 4-24 | % | 178 | 48 | 77 | 99 | 63 | 78 | | 12 |
| Glmnet | 4-5 | Log10 | 198 | 48 | 16 | 88 | 60 | 17 | | 69 |
| RF | 4-5 | Log10 | 198 | 48 | 16 | 99 | 82 | 0 | | 97 |
| LogitBoost | 4-5 | Log10 | 198 | 48 | 16 | 92 | 74 | 17 | | 84 |
| Glmnet | 4-6 | Log10 | 198 | 48 | 36 | 76 | 59 | 21 | | 80 |
| RF | 4-6 | Log10 | 198 | 48 | 36 | 100 | 56 | 0 | | 88 |
| LogitBoost | 4-6 | Log10 | 198 | 48 | 36 | 89 | 54 | 50 | | 56 |
| Glmnet | 4-10 | Log10 | 197 | 48 | 63 | 77 | 62 | 75 | | 40 |
| RF | 4-10 | Log10 | 197 | 48 | 63 | 100 | 69 | 92 | | 33 |
| LogitBoost | 4-10 | Log10 | 197 | 48 | 63 | 97 | 67 | 83 | | 40 |
| Glmnet | 4-24 | Log10 | 178 | 48 | 77 | 90 | 57 | 70 | | 12 |
| RF | 4-24 | Log10 | 178 | 48 | 77 | 100 | 77 | 96 | | 12 |
| LogitBoost | 4-24 | Log10 | 178 | 48 | 77 | 99 | 63 | 78 | | 12 |
