# Supplementary material for: The Influence of FUT2 and FUT3 Polymorphisms and Nasopharyngeal Microbiome on Respiratory Infections in Breastfed Bangladeshi Infants from the Microbiota and Health Study
Source: mSphere. 2021 Nov 10;6(6):e00686-21. doi: 10.1128/mSphere.00686-21 (PMC8579893; doi:10.1128/mSphere.00686-21)
Supplement: TABLE S4 [file msphere.00686-21-st004.docx]

|  | **2 months** | | **4 months** | |
| --- | --- | --- | --- | --- |
|  | **Culture undetected** | **Culture detected** | **Culture undetected** | **Culture detected** |
|  | *Haemophilus influenzae* (MGS021) | | | |
| MGS > 0 | 90 | 67 | 68 | 90 |
| MGS = 0 | 47 | 1 | 24 | 0 |
|  | *Moraxella catarrhalis* (MGS018) | | | |
| MGS > 0 | 70 | 70 | 73 | 71 |
| MGS = 0 | 63 | 2 | 36 | 2 |
|  | *Streptococcus pneumoniae* (MGS008) | | | |
| MGS > 0 | 88 | 113 | 57 | 123 |
| MGS = 0 | 3 | 0 | 2 | 0 |
