# Supplementary material for: The Influence of FUT2 and FUT3 Polymorphisms and Nasopharyngeal Microbiome on Respiratory Infections in Breastfed Bangladeshi Infants from the Microbiota and Health Study
Source: mSphere. 2021 Nov 10;6(6):e00686-21. doi: 10.1128/mSphere.00686-21 (PMC8579893; doi:10.1128/mSphere.00686-21)
Supplement: Supplementary file 8 [file msphere.00686-21-sf001.docx]

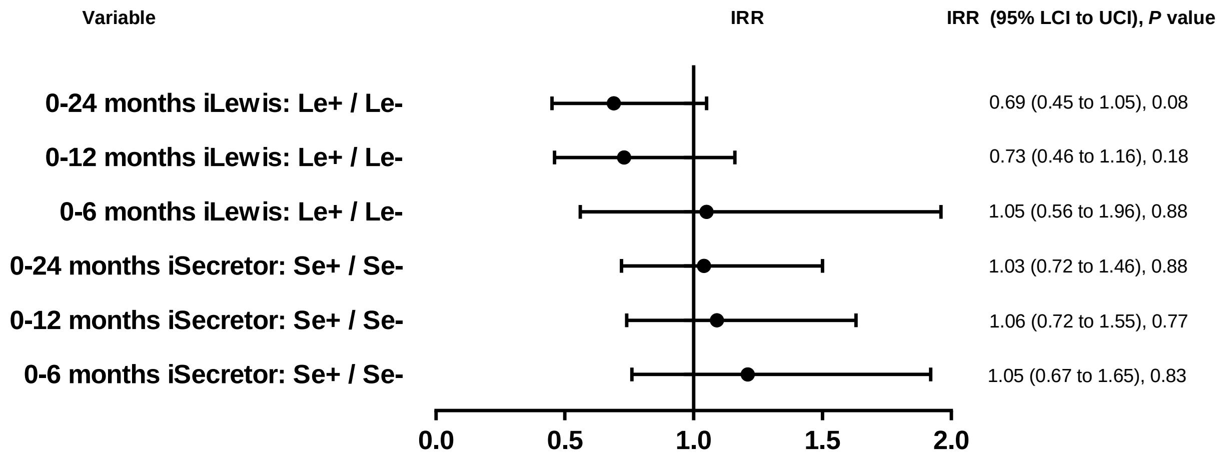
Variable
IRR
IRR (95% LCI to UCI), *P* value
0-24 months iLewis: Le+ / Le-
0-12 months iLewis: Le+ / Le-
0-6 months iLewis: Le+ / Le-
0-24 months iSecretor: Se+ / Se-
0-12 months iSecretor: Se+ / Se-
0-6 months iSecretor: Se+ / Se-
0.69 (0.45 to 1.05), 0.08
0.73 (0.46 to 1.16), 0.18
1.05 (0.56 to 1.96), 0.88
1.03 (0.72 to 1.46), 0.88
1.06 (0.72 to 1.55), 0.77
1.05 (0.67 to 1.65), 0.83
0.0
0.5
1.0
1.5
2.0
